# Complex Interplay among DNA Modification, Noncoding RNA Expression and Protein-Coding RNA Expression in *Salvia miltiorrhiza* Chloroplast Genome

**DOI:** 10.1371/journal.pone.0099314

**Published:** 2014-06-10

**Authors:** Haimei Chen, Jianhui Zhang, George Yuan, Chang Liu

**Affiliations:** 1 IMPLAD PacBio Advanced Genomic Analysis Laboratory, Institute of Medicinal Plant Development, Chinese Academy of Medical Sciences & Peking Union Medical College, Haidian District, Beijing, P. R. China; 2 Pacific Biosciences, Menlo Park, California, United States of America; Universidade Federal do Rio Grande do Sul, Brazil

## Abstract

*Salvia miltiorrhiza* is one of the most widely used medicinal plants. As a first step to develop a chloroplast-based genetic engineering method for the over-production of active components from *S. miltiorrhiza*, we have analyzed the genome, transcriptome, and base modifications of the *S. miltiorrhiza* chloroplast. Total genomic DNA and RNA were extracted from fresh leaves and then subjected to strand-specific RNA-Seq and Single-Molecule Real-Time (SMRT) sequencing analyses. Mapping the RNA-Seq reads to the genome assembly allowed us to determine the relative expression levels of 80 protein-coding genes. In addition, we identified 19 polycistronic transcription units and 136 putative antisense and intergenic noncoding RNA (ncRNA) genes. Comparison of the abundance of protein-coding transcripts (cRNA) with and without overlapping antisense ncRNAs (asRNA) suggest that the presence of asRNA is associated with increased cRNA abundance (*p*<0.05). Using the SMRT Portal software (v1.3.2), 2687 potential DNA modification sites and two potential DNA modification motifs were predicted. The two motifs include a TATA box–like motif (CPGDMM1, “TATANNNATNA”), and an unknown motif (CPGDMM2 “WNYANTGAW”). Specifically, 35 of the 97 CPGDMM1 motifs (36.1%) and 91 of the 369 CPGDMM2 motifs (24.7%) were found to be significantly modified (*p*<0.01). Analysis of genes downstream of the CPGDMM1 motif revealed the significantly increased abundance of ncRNA genes that are less than 400 bp away from the significantly modified CPGDMM1motif (*p*<0.01). Taking together, the present study revealed a complex interplay among DNA modifications, ncRNA and cRNA expression in chloroplast genome.

## Introduction

*Salvia miltiorrhiza*, also known as Chinese sage, tan shen, or danshen, is highly valued in traditional Chinese medicine for its roots or rhizomes [1]. *S. miltiorrhiza* extracts are the main components of two of the ten best-selling traditional Chinese medical products, each with a total sale of around 100 million US dollars. To date, more than 70 active components have been isolated from *S. miltiorrhiza* [2], [3]. These active ingredients can be classified into two groups: lipid-soluble tanshinones and water-soluble phenolic acids. Compounds from both groups exhibit antioxidant, antitumor, anti-inflammatory, and antimicrobial properties [1].

Given the pharmacological and economic importance of the active components of *S. miltiorrhiza*, their overproduction remains an active research area. Recent studies have shown that chloroplasts are ideal hosts for the expression of genes related to the production of secondary metabolites [4] because these organelles support (1) the accumulation of large amounts of protein; (2) transgene stacking because of the presence of polycistron; (3) proper protein folding; (4) site-specific integration; and (5) maternal inheritance, which prevents the dissemination of transgenes through pollen. However, a detailed understanding of the chloroplast genome structure, gene contents, and gene expression regulation is a prerequisite for the development of effective chloroplast-based transgenic systems.

Chloroplasts are plant-specific organelles for photosynthesis, starch storage, nitrogen metabolism, sulfate reduction, fatty acid synthesis, DNA and RNA synthesis, and the synthesis of 50% of soluble protein in leaves [5]. By March 2014, 442 complete Viridiplantae chloroplast genomes are deposited in GenBank. Comprehensive reviews have been conducted on the (1) structure and biology of chloroplast genomes [6]; (2) RNA metabolism and translation regulation [7]; and (3) the coordination of gene expression between organellar and nuclear genomes [8]. Although the *S. miltiorrhiza* chloroplast genome has been recently assembled and analyzed [9], the expression profile, as well as the presence and functions of noncoding RNA (ncRNA), and DNA modifications have not been studied in depth. Thus, the current study aims to clarify these issues.

ncRNAs are RNAs that do not encode proteins, but have multiple functions. ncRNAs can be classified into two broad categories: house-keeping and regulatory. House-keeping ncRNA, which include ribosomal, small nuclear, small nucleolar, and transfer RNA (tRNA), is constitutively expressed [10]. On the other hand, regulatory ncRNA are often expressed during specific developmental stages or under particular nutritional or environmental conditions [11]. One ncRNA subset, called natural antisense transcripts (NATs, also called asRNA), are endogenous RNA molecules with regions complementary to a sense transcript (cRNA). Through base-pairing with their targets, these asRNA elicit translational inactivation/activation, mRNA stabilization/destabilization, or differential transcription termination [12], [13].

Previous studies have identified dozens of ncRNA in chloroplasts [10], [14]. A number of studies have elucidated the regulatory roles of chloroplast ncRNA. For the first example, an *ndh*B asRNA covers two editing sites of the *ndh*B gene and a group II intron splice acceptor site, and might play important role in RNA maturation or stability [14]. For the second example, two different antisense RNAs of *psbT* gene were found to form double-stranded RNA/RNA hybrids, which results in translational inactivation of the *psbT* mRNA. The hybrid was further hypothesized to provide protection against nucleolytic degradation of mRNA during photo-oxydative stress conditions [15]. For the third example, antisense RNA of *rbc*L and *atp*B genes form stable double-stranded molecules with sense strand transcripts and prevents their translation [16]. For the forth example, the expression of complementary RNA of *Rpo*B-2 from chloroplast transgenes affects editing efficiency of transgene and other endogenous chloroplast transcripts [17]. For the last example, overexpression of AS5, a chloroplast-encoded asRNA complementary to the 5S rRNA in tobacco, destabilizes 5S rRNA and retards plant growth [18]. These findings suggest that the ncRNA in the chloroplast genome has various regulatory roles. Therefore, identifying and characterizing the ncRNA in the *S. miltiorrhiza* chloroplast genome would help reveal the functions of this important group of gene expression regulators.

DNA contains a large variety of functionally important modifications [19]. The most common modification, DNA methylation, involves the addition of a methyl group to cytosine or adenine DNA nucleotides by methyl transferase (MTase). The most common methylation types are N6-methyladenine (m6A), N4-methylcytosine (m4C), and 5-methylcytosine (m5C) [20]. In plants, DNA methylation is essential for growth and development, and it affects gene expression, genomic imprinting, heterochromatin assembly, and protection of the genome against migrating transposable elements [21], [22]. It should be pointed out that most studies on the effect of DNA modifications on gene expression focus on the nuclear DNAs. For chloroplast DNAs, Ahlert et al. [23] observed the insensitivity of plastid gene expression to both adenine and cytosine methylation of the plastid DNA by expressing two cyanobacterial DNA methyltransferase genes from the tobacco plastid genome. However, whether or not these observations reflect the natural stages of DNA modifications in chloroplast genomes is unknown. As a result, identification of potential DNA modification sites in the *S. miltiorrhiza* chloroplast genome would provide valuable information on the epigenetic regulation of its gene expression.

In this study, we used the Single-Molecule Real-Time (SMRT) Sequencing and strand-specific RNA-Seq technologies to characterize in detail the *S. miltiorrhiza* chloroplast genome, transcriptome, and DNA modifications. Detailed analyses of DNA modifications as well as of ncRNA and cRNA expression suggest a complex interplay between these processes. The results of this study would provide valuable information for the construction of chloroplast-based transgenic systems, as well as lay the foundation for research on ncRNA and DNA modifications in regulating the expression of coding genes in chloroplasts from other organisms.

## Materials and Methods

### Materials and DNA sequencing

Fresh leaves from three plants of *S. miltiorrhiza* Bunge *cv* 99-3 were collected from the Institute of Medicinal Plant Development, Beijing, China. The leaves are pooled together and total DNA was extracted from the leaves using a plant genomic DNA kit (Tiangen, China). The genomic DNA of *S. miltiorrhiza* were isolated and subjected to DNA sequencing using SMRT sequencing, a parallelized single molecule DNA sequencing by synthesis technology developed by PacBio (Pacific Biosciences, Menlo Park, California, United States of America). The DNA sequencing is done on a SMRTcell containing hundreds thousand of zero-mode waveguide (ZMW) nano-pores. Inside each ZMW, a single active DNA polymerase with a single molecule of single stranded DNA template is immobilized to the bottom through which light can penetrate and create a visualization chamber. The nucleotide substrate is phosphor-linked to a fluorescence dye. As the DNA synthesis proceeds, the fluorescence signal is detected when a nucleotide is held by the DNA polymerase before the nucleotide being incorporated into the DNA strand, resulting in the DNA sequencing in real time. We choose SMRT technology for this study as it is the only technology that is currently capable to directly detect potential modifications on native DNA without PCR amplification [24], The DNA was used to construct two libraries with the insert sizes of 1 kb and 10 kb, which were subjected to DNA sequencing following the standard protocol provided by the manufacturer (PacBio).

### Genome Assembly

To assemble the genome, we downloaded 277 chloroplast genomes from GenBank on October 2012. These sequences were used to search against PacBio reads for *S. miltiorrhiza*. Similar reads with an E value < 1e-5 were extracted and subjected to sequence assembly using Seqman (version 9.0). The genome sequence of *Sesamum indicum*, which has the highest overall sequence similarity with *S. miltiorrhiza* reads, was used as a reference to order the contigs. The gaps were filled by iteratively searching the PacBio *S. miltiorrhiza* reads using the contigs, extracting reads that share sequence similarities, extending the ends, adding the reads to the assembly, and then performing reassembly. After obtaining an initial assembly, we designed primers (Table S1 in File S1) to amplify the four junctions between the single-copy segments and the inverted repeats. We sequenced the PCR products using a BigDyeV3.1 Terminator Kit for 3730XL (Life Technologies) and assembled high-quality Sanger sequences into the initial assembly to obtain the final assembly using Seqman (DNASTAR, WI). The final assembly has been deposited into EMBL (accession number HF586694). Note that another chloroplast genome of *S. miltiorrhiza* was published during the duration of this study [9]. After comparison, only 41 base differences were found between the two genome assemblies; the general features are otherwise identical (results not shown).

### RNA extraction and RNA-Seq analysis

Total RNA was extracted from fresh leaves of three *S. miltiorrhiza* plants using an RNAprep pure plant kit (Tiangen, China) separately following the manufacturer's recommendation. The three RNA samples were pooled and the cDNA was amplified using a random-priming protocol. Ribosomal RNA was removed from total RNA using MICROB*Express* Bacterial mRNA Enrichment Kit (Ambion, AM1905). A RNA-Seq library was constructed following a strand-specific protocol (dUTP, Illumina) [25]. The resulting cDNA were cleaved into small fragments (300 bp to 400 bp) to construct sequencing libraries, followed by emulsion PCR and then sequenced according to the manufacturer's protocols. The resulting data can be found in GenBank with accession number SRA1045051. All reads were mapped to the chloroplast genome assembly using Tophat (v1.3.2) [26]. The polycistrons and ncRNA were identified manually based on the identification of contigs assembled from overlapping RNA-Seq reads along the *S. miltiorrhiza* genome assembly. The 5′ and 3′ ends were determined based on the very ends of each contig. The RNA-Seq data were visualized and quantified using SeqMonk (v0.23.0, Babraham Bioinformatics, UK) and Tablet (v1.12.12.05) [27]. SeqMonk is able to call the abundances/expression levels for given regions and output the abundance as number of Reads Per Million (RPM). Considering the low expression levels of ncRNA, no coverage cutoff was set. In another word, the minimal coverage is set as 1 for the RNA-Seq data analysis.

### Strand-specific real-time quantitative PCR

Total RNA from leaves of three plants were extracted as described above. Strand-specific real-time quantitative PCR (ss-qPCR) was performed as described previously [28]. Briefly, twenty-four pairs of antisense ncRNA and cRNA were randomly selected, and ss-qPCR primers were designed (Table S2 in File S1). A sequence similarity search was conducted to ensure that the primer sequences would uniquely amplify the expected regions. Strand specific cDNA templates were synthesized with tagged primers to add 18 to 20 nucleotide tags unrelated to S. *miltiorrhiza* (Table S2 in File S1: cRNAtag; GCTAGCTTCAGCTAGGCATC and mRNAtag; CCAGATCGTTCGAGTCGT) at the 5′ end (Table S2 in File S1) [28]. Reverse transcription with the tagged primer was performed using a PrimeScript RT-PCR Kit (Takara). The PCR was performed as follows: a 10 µl mixture containing approximately 500 ng of total RNA, 1 µl of dNTP mixture (10 mM each), and 2 pmol of tagged primer was heated for 5 min at 65°C and then cooled to 4°C. The reaction mixture contained 4 µl of PrimeScript buffer (5×), 0.5 µl of RNase inhibitor (40 U/µl), 0.5 µl of PrimeScript RTase, and 5 µl of RNase-free water. The RT reaction was conducted at 45°C for 30 min and terminated by heating at 70°C for 15 min. The 0.5 µl reverse-transcription product was amplified with an SYBR Premix Ex Taq kit (Takara) according to manufacturer's instructions. The qPCR experiment was performed on an ABI 7500 Fast instrument (Applied Biosystems). The cycle conditions were as follows: 95°C for 10 min, followed by 40 cycles of 95°C for 5 s, and 60°C for 34 s. The primers used are listed in Table S2 in File S1. Melting curve acquisition and analysis were performed immediately after amplification using default settings, which are 95°C for 15 sec, followed by 60°C for 1 min, and 95°C for 15 sec.

### Validation of RNA-Seq results with ss-qPCR

To validate the strand specificity and expression abundance of ncRNA detected in our RNA-Seq data, three total RNA samples extracted from leaves of three *S. miltiorrhiza* plants were analyzed using ss-qPCR as described above. The expression quantification by qPCR was performed as described previously [29] and a cRNA coding for *atp*B gene was chosen as the internal control for each ss-qPCR experiment.

For RNA-Seq data, we first specified the start and end of the overlapping regions for each pair of ncRNA and cRNA. SeqMonk was used to calculate the RPMs for ncRNA and cRNA for the overlapping regions. Validation of differential gene expression between ncRNA and cRNA was performed as previously described [30], [31]. Briely, the log fold change, or log ratio, of ncRNA and cRNA was calculated as log[(RPM for ncRNA)/(RPM for cRNA)]. For ss-qPCR data, the log ratio for each technical replication was calculated as [(40-ct) for ncRNA] – [(40-ct) for cRNA]. The log ratios of the three technical replications were averaged to create the mean and standard deviation of log ratio for each biological replicate. The statistical significance between the RNA-Seq and ss-qPCR results was tested using one-sample *t* test.

### Detection of DNA modifications and identification of DNA modification motifs

The total DNA was sequenced using SMRT technology following the standard procedures as described above. Sequence preprocessing and DNA modification detection were performed using the SMRT Analysis pipeline (version 1.3.2). The DNA modification motifs were identified using MotifFinder (PacBio).

### Statistical Analysis

Statistical analyses, including one-sample *t* test and analysis of variance, were conducted using the JMP software (v9.0, SAS, NC) and customer scripts written with Perl module Statistics::Distributions (v1.02).

## Results

### Integrative analyses of the *S. miltiorrhiza* chloroplast

**Figure 1:**
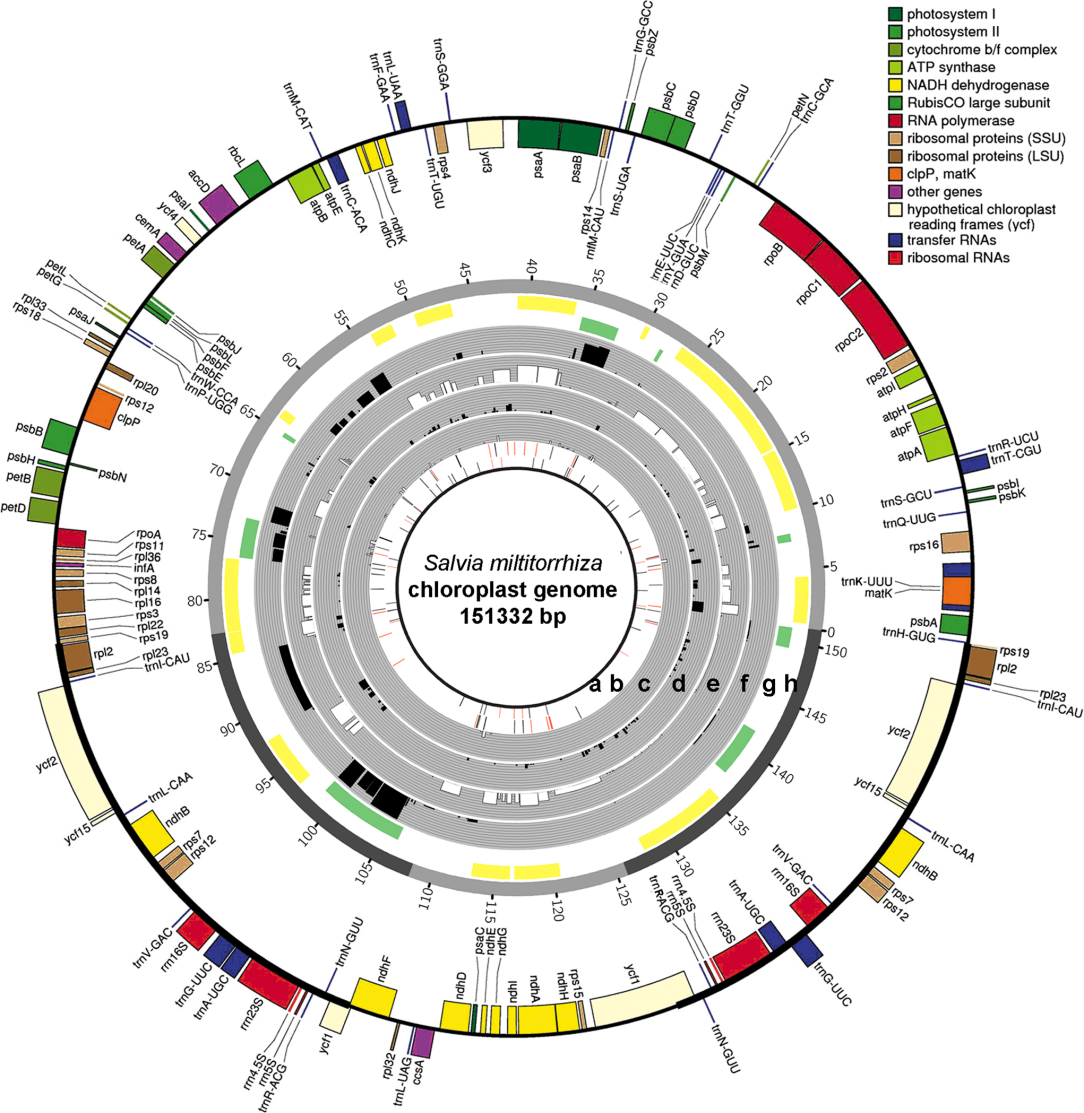
Ideogram showing the transcriptome and DNA modifications of the *Salvia miltiorrhiza* chloroplast genome. The predicted genes are drawn on the outermost circle. Those drawn outside the circle are transcribed in a clockwise direction, whereas those drawn inside the circle are transcribed in a counterclockwise direction. The distributions of DNA modification motif 1 on the positive strand (a) and the negative strand (b) are shown as bars. The red bar indicates motifs that are significantly modified. The expression levels of the noncoding genes on the positive (c) and negative strands (d); the coding genes on the positive (e) and negative strands (f) are shown in black and white arcs on grey background, The polycistrons on the positive (g) and negative strands (h) are shown as green and yellow arcs on white background. The heights of the arcs in (c) to (h) represent the expression levels of the corresponding transcripts.

We used strand-specific RNA-Seq, and SMRT technologies (Figure S1 in File S1) to characterize the genome, transcriptome, and DNA modifications of the *S. miltiorrhiza* chloroplast. An ideogram summarizing our results is shown in Figure 1, which include the following information: the putative DNA modification sites on the positive strands (a) and negative strands (b); the expression level of noncoding genes on the positive strands (c) and negative strand (d); the expression level of coding genes on the positive strands (e) and negative strand (f); and the identified polycistrons on the positive strands (g) and negative strands (h). This ideogram provides an overall picture of the transcriptome and DNA modifications in the *S. miltiorrhiza* chloroplast, which are described in detail below.

### Analysis of chloroplast gene expression in leaves using RNA-Seq technology

We conducted strand-specific RNA-Seq analysis of the *S. miltiorrhiza* transcriptome to understand the gene expression landscape of the *S. miltiorrhiza* genome. Chloroplast genes are of prokaryotic origin. Therefore, we used a protocol designed to analyze prokaryotic transcriptome. We generated up to 53,849,024 read pairs. Using Tophat [26], 553,014 (1.0%) of the reads were mapped to the *S. miltiorrhiza* chloroplast genome. In a previous study, 133 genes have been identified in the *S. miltiorrhiza* chloroplast genome [9], including 131 predicted functional genes and 2 pseudogenes. The functional genes consist of 114 unique genes, including 80 protein-coding genes, 30 tRNA genes, and 4 rRNA molecules. For genes with more than one copy, we selected only one for further analysis. Except for 14 unique genes, all genes were expressed, including 80 protein-coding genes, 4 rRNA genes, and 16 tRNA genes (Table 1). Fourteen tRNA were not found expressed. One possibility is that the tRNA transcript lengths range from 70 bp to 92 bp. These transcripts were excluded from our library because only fragments that are 300 bp to 400 bp long were selected to construct the library. By contrast, 16 tRNA genes were expressed, 9 of which were embedded in polycistrons (Table 1) and 3 have large introns. The remaining four tRNA coding genes (*trn*F-GAA, *trn*N-GUU, *trn*S-GGA, and *trn*Q-UUG) were embedded in long noncoding transcripts (see below).

**Table 1:** **Expression abundance of all coding genes and polycistrons determined using RNA-Seq analysis.**

Gene Name^a^	Start	End	Strand	Arbitrary Log Abundance	Gene Name	Start	End	Strand	Arbitrary Log Abundance
^1^ *psb*A	392	1450	N^b^	12.93	*psb*B	71218	72744	P	14.08
^1^ *trn*K-UUU	1672	4266	N	6.90	^13^ *psb*T	72921	73028	P	1.85
^1^ *mat*K	1972	3534	N	5.76	*psb*N	73089	73220	N	5.76
*rps*16	4834	5944	N	5.85	^13^ *psb*H	73308	73547	P	5.56
^2^ *psb*K	7380	7559	P^b^	8.21	^13^ *pet*B	73673	75022	P	8.88
^2^ *psb*I	7905	8066	P	5.18	^13^ *pet*D	75216	76418	P	7.88
*trn*G-UCC	8923	9677	P	3.85	^14^ *rpo*A	76595	77602	N	5.76
^3^ *atp*A	10042	11565	N	9.67	^14^ *rps*11	77674	78090	N	6.44
^3^ *atp*F	11661	12913	N	7.96	^14^ *rpl*36	78200	78313	N	3.85
^3^ *atp*H	13187	13432	N	8.74	^14^ *inf*A	78409	78642	N	5.18
^3^ *atp*I	14403	15146	N	5.66	^14^ *rps*8	78769	79173	N	5.94
^4^ *rps*2	15374	16084	N	7.02	^14^ *rpl*14	79363	79731	N	5.31
^4^ *rpo*C2	16293	20480	N	6.98	^14^ *rpl*16	79863	81143	N	7.06
^4^ *rpo*C1	20638	23451	N	7.69	^14^ *rps*3	81279	81941	N	6.66
^4^ *rpo*B	23478	26690	N	7.06	^14^ *rpl*22	81926	82393	N	6.31
^5^ *trn*C-GCA	27834	27904	P	1.70	^14^ *rps*19	82463	82741	N	3.85
^5^ *pet*N	28061	28156	P	2.85	^15^ *rpl*2	82801	84283	N	9.38
*psb*M	29133	29237	N	4.85		149748	151230	P	
^6^ *trn*D-GUC	29758	29831	N	1.85	^15^ *rpl*23	84302	84583	N	7.06
^6^ *trn*Y-GUA	29942	30025	N	1.85		149448	149729	P	
^6^ *trn*E-UUC	30097	30169	N	1.70	*ycf*2	84911	91762	P	7.38
^7^ *psb*D	32159	33220	P	14.92		142269	149120	N	
^7^ *psb*C	33168	34589	P	13.64	^16^ *trn*L-CAA	92362	92442	N	1.70
^7^ *psb*Z	35262	35450	P	8.62		141589	141669	P	-
^8^ *rps*14	36188	36490	N	10.09	^16^ *ndh*B	93002	95155	N	12.80
^8^ *psa*B	36613	38817	N	12.50		138876	141029	P	-
^8^ *psa*A	38843	41095	N	12.58	^16^ *rps*7	95484	95951	N	12.59
*ycf*3	41886	43817	N	9.64		138080	138547	P	-
*trn*S-GGA	44634	44720	P	3.44	^16^3′-*rps*12	96005	96785	N	10.72
*rps*4	44997	45602	N	6.66		137246	138026	P	-
^9^ *trn*L-UAA	46728	47267	P	5.02	^17^ *rrn*16S	98690	100180	P	18.34
^9^ *trn*F-GAA	47566	47638	P	2.85		133851	135341	N	-
^9^ *ndh*J	48171	48647	N	6.94	^17^ *trn*I-GAU	100479	101490	P	13.16
^9^ *ndh*K	48758	49435	N	9.43		132541	133552	N	-
^9^ *ndh*C	49487	49849	N	8.90	^17^ *trn*A-UGC	101555	102422	P	13.82
*trn*V-UAC	50799	51447	N	5.66		131609	132476	N	-
^10^ *atp*E	51904	52305	N	5.66	^17^ *rrn*23S	102581	105397	P	18.79
^10^ *atp*B	52302	53798	N	9.76		128634	131450	N	-
*rbc*L	54563	56017	P	13.52	^17^ *rrn*4.5S	105497	105599	P	7.56
*acc*D	56708	58195	P	8.57		128432	128534	N	-
*psa*I	58658	58768	P	1.85	^17^ *rrn*5S	105824	105954	P	2.85
*ycf*4	59224	59778	P	5.31		128077	128207	N	-
*cem*A	60326	61015	P	5.44	^17^ *trn*R-ACG	106188	106261	P	3.85
*pet*A	61221	62183	P	6.66		127770	127843	N	-
^11^ *psb*J	63245	63367	N	4.44	*ndh*F	108206	110422	N	7.85
^11^ *psb*L	63497	63613	N	6.56	*rpl*32	110875	111045	P	1.85
^11^ *psb*F	63637	63756	N	7.56	*ccs*A	111907	112884	P	2.85
^11^ *psb*E	63771	64022	N	9.60	^18^ *ndh*D	113116	114630	N	9.29
^12^ *pet*L	64829	64924	P	1.70	^18^ *psa*C	114766	115011	N	5.31
^12^ *pet*G	65103	65216	P	1.85	^18^ *ndh*E	115262	115567	N	6.76
*psa*J	65939	66073	P	5.66	^18^ *ndh*G	115783	116313	N	7.88
*rps*18	66890	67195	P	2.85	^19^ *ndh*I	116689	117195	N	7.35
*rpl*20	67427	67813	N	4.18	^19^ *ndh*A	117276	119352	N	9.72
5′-*rps*12	68623	68736	N	1.85	^19^ *ndh*H	119354	120535	N	7.31
*clp*P	68860	70769	N	6.71	*ycf*1	121333	126849	N	4.66

a The superscript number in front of each gene name indicate the polycistron number.

b N: negative; P: positive.

These genes were quantified using the Seqmonk software. The results are shown in Table 1. The most highly expressed genes were the rRNA 23S and rRNA 16S, as well as *psb*A, *psb*B, *psb*C, *psb*D, and *rbc*L. This result is consistent with the involvement of rRNA in protein synthesis as well as with the participation of *psb* genes and *rbc*L in photosynthesis. Interestingly, two tRNA genes, namely, *trn*A-UGC and *trn*I-UUC, were highly expressed at levels comparable to those of rRNA genes and *psb* subunit genes. The *trn*A-UGC and *trn*I-GAU transcripts accounted for 60.60% and 38.18% of all tRNA transcripts, respectively, even though alanine and isoleucine only account for 2/30 (6.7%) of the amino acids in the *S. miltiorrhiza* genome. Detailed examination showed that these two tRNA genes are located in polycistron pc17, which contains the rRNA genes. Their expression levels are consistent with those of other genes in the same polycistron. Therefore, this region is a potential site for foreign gene insertion (see below).

### Polycistron identification

Most genes from chloroplast genomes are transcribed in polycistrons [32]. We determined the polycistrons in the *S. miltiorrhiza* chloroplast genome based on the presence of a continuous transcript. In total, we found 19 polycistronic transcripts containing 71 genes, which include 58 coding-protein genes, 9 tRNA, and all 4 rRNA. The polycistron identity, its member genes, and the strand on which the gene is located are shown in Table 1. By comparing with the results reported by Yang et al. [33], the polycistron (pc1) was identified for the first time. The transcripts for a number of genes (*atp*I, *rpo*C2) were not entirely covered by the RNA-Seq reads. However, based on the results of a previous study [33], we consider these cases as examples of biased coverage of the sequencing technologies. Moreover, the corresponding broken transcripts (polycistron pc3) were treated as a continuous polycistronic transcript.

### ncRNA identification and classification

**Figure 2:**
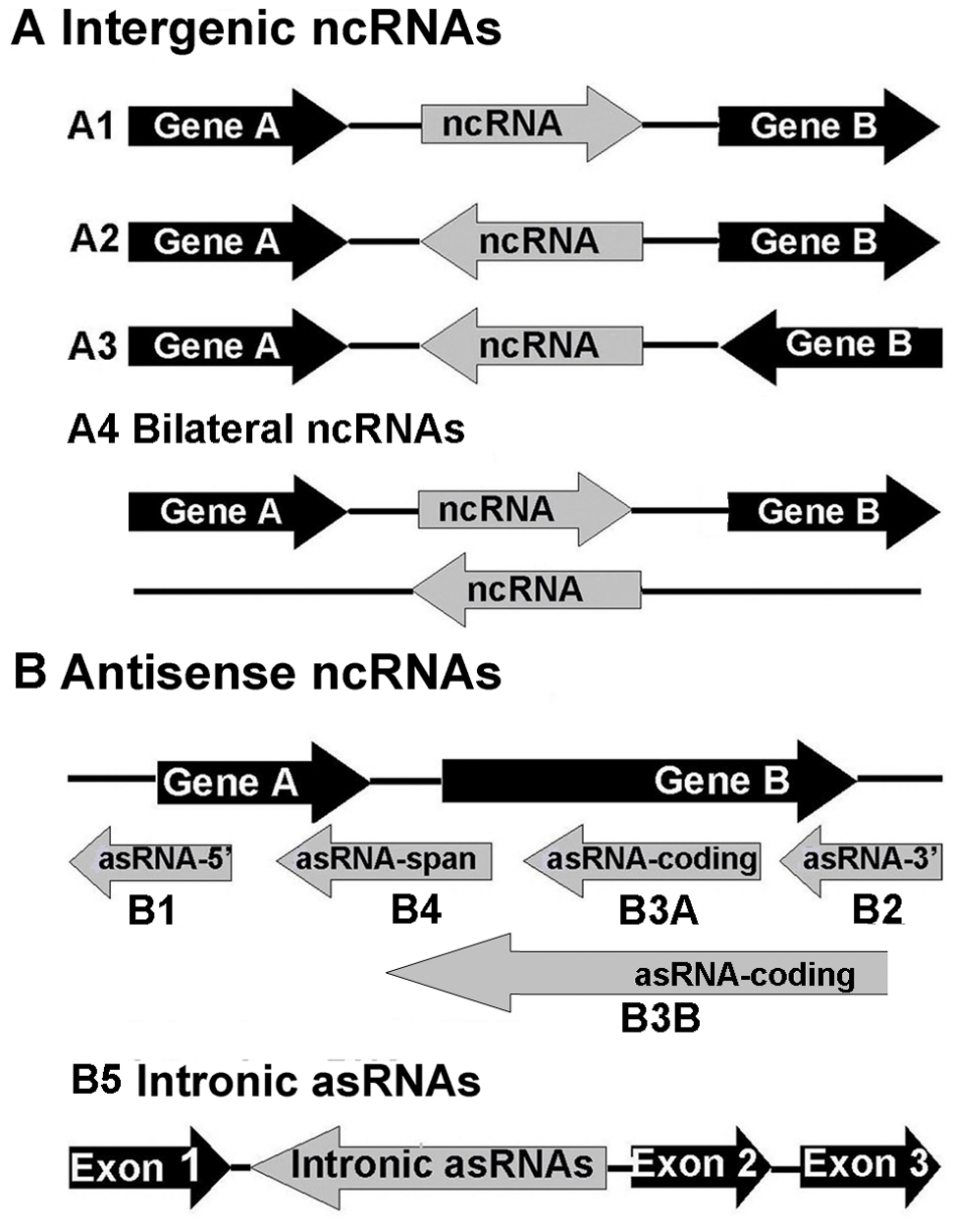
Schematic representations of various types of ncRNA identified in this study. Protein-coding genes and their exons are presented in black, whereas ncRNA are presented in grey. The arrows indicate the direction of the transcripts. (A) Intergenic ncRNA, ncRNA expressed in the intergenic region. Four different types, namely, A1, A2, and A3, A4 are shown. (B) Antisense ncRNA, ncRNA transcribed from the antisense strand of protein-coding genes. This ncRNA overlaps with either the 5′ (B1) end or 3′ (B2) end of the coding region, is embeded in (B3A) or spans (B3B) one entire protein-coding gene, or spans two protein-coding genes (B4). Intronic asRNA (B5) is transcribed entirely from an intron of a coding gene.

A recent study found unexpected diversity in the ncRNA in the chloroplast transcriptome [10]. We performed a systematic analysis to identify ncRNA in the chloroplast genome of *S. miltiorrhiza*. To be consistent, we defined coding RNA (cRNA) as any RNA transcripts transcribed from genes encoding proteins. We define non-coding RNA (ncRNA) as any RNA transcripts transcribed that do not encode proteins and have a length > = 100 bp. We identified 136 ncRNA transcripts based on these criteria (Table 2). These ncRNAs are classified into two groups, namely, intergenic ncRNA (Figure 2A) and antisense ncRNA (asRNA, Figure 2B), based on their positions relative to the cRNA genes. Intergenic ncRNA are those located between two transcripts or polycistrons and its distance to the start or end position of each transcript was at least 100 bp (Figure 2A). Three types, namely, A1, A2, and A3, were found. A special case of intergenic ncRNA expresses from both strands and was defined as bilateral ncRNA (A4, Figure 2A).

**Table 2:** **List of ncRNAs identified in the** ***S. miltiorrhiza*** **genome.**

ID	Type of ncRNA^a^	Corresponding protein-coding RNA (cRNA)	Start	End	Strand	Arbitrary Log Abundance		Log Abundance (ncRNA/cRNA)
						ncRNA	cRNA	
nc1	B3B	*psb*A	200	1495	P^b^	8.75	12.95	−4.2
nc2	B3A	*mat*K	2165	2310	P	1.7	3.85	−2.15
nc3	B5	*trn*K-UUU	3257	3927	P	3.85	5.31	−1.46
nc4	B1	*trn*K-UUU	4062	4338	P	4.18	4.44	−0.26
nc5	B3B	*trn*Q-UUG	6617	7053	P	7.64	1.7	5.93
nc6	B4	*psb*K-*psb*I	7413	7869	N^b^	5.02	9.37	−4.35
nc7	B1	*trn*S-GCU	8278	8541	P	4.18	1.7	2.47
nc8	B3A	*atp*A	10264	10519	P	2.85	6.38	−3.52
nc9	B3A	*atp*A	10960	11171	P	2.85	8.65	−5.79
nc10	B2	*atp*H	13082	13272	P	1.85	8.44	−6.58
nc11	B1	*atp*H	13288	13456	P	1.7	8.45	−6.75
nc12	A2	*atp*H-*atp*I	13547	13720	P	1.7	4.66	−2.96
nc13	B2	*rpo*C2	16319	16513	P	1.85	1.85	0
nc14	B3A	*rpo*C2	16624	16832	P	2.85	4.18	−1.32
nc15	B3A	*rpo*C2	17149	17355	P	1.85	1.85	0
nc16	B3A	*rpo*C2	18119	18313	P	1.85	1.7	0.15
nc17	B3A	*rpo*C2	19678	19892	P	1.85	2.85	−1
nc18	B3A	*rpo*C1	21473	21691	P	1.85	3.85	−2
nc19	B3B	*pet*N	27966	28380	N	3.44	5.18	−1.74
nc20	A3	*pet*N-*psb*M	28478	28711	N	4.44	6.85	−2.42
nc21	A3	*pet*N-*psb*M	28793	28948	N	2.85	5.44	−2.58
nc22	B4	*trn*D-GUC-*trn*E-UUC	29689	30029	P	4.18	4.18	0
nc23	A2	*trn*T-GGU-*psb*D	31586	31743	N	1.7	7.66	−5.96
nc24	B3A	*psb*D	32467	32646	N	1.85	12.95	−11.09
nc25	B3A	*psb*D	32730	32891	N	1.7	11.1	−9.39
nc26	B3A	*psb*C	33317	33469	N	1.7	11.64	−9.94
nc27	B3A	*psb*C	33534	33703	N	1.7	9.7	−8
nc28	B3A	*psb*C	34011	34229	N	3.44	11.47	−8.03
nc29	B3B	*trn*G-GCC	35562	35803	N	3.44	1.7	1.74
nc30	B4	*rps*14-*psa*B	36400	36644	P	3.44	10.13	−6.69
nc31	B3A	*psa*B	36801	36967	P	1.7	9.52	−7.82
nc32	B3A	*psa*B	37108	37418	P	1.85	8.3	−6.44
nc33	B3A	*psa*B	37587	37873	P	1.85	10.96	−9.1
nc34	A2	*psa*A-*ycf*3	41278	41422	P	1.7	6.61	−4.91
nc35	B2	*ycf*3	41854	42231	P	5.85	7.35	−1.49
nc36	B5	*ycf*3	42290	42563	P	5.02	8.16	−3.13
nc37	B1	*ycf*3	43721	43913	P	1.85	7.47	−5.61
nc38	A4	*ycf*3-*trn*S-GGA	43994	44309	N	5.02	3.58	1.44
nc39	A4	*ycf*3-*trn*S-GGA	44037	44246	P	4.18	4.52	−0.34
nc40	A3	*ycf*3-*trn*S-GGA	44256	44483	P	1.85	2.94	−1.09
nc41	B3A	*rps*4	45313	45558	P	1.85	5.76	−3.91
nc42	A3	*trn*T-UGU-*trn*L-UAA	46416	46572	N	1.85	1.7	0.15
nc46	B2	*ndh*J	47866	48115	P	1.85	5.15	−3.3
nc47	B3A	*ndh*J	48199	48592	P	4.18	6.81	−2.63
nc48	B3A	*ndh*K	49174	49332	P	1.85	8.54	−6.69
nc49	B4	*ndh*K-*ndh*C	49508	49643	P	1.7	7.83	−6.13
nc50	A2	*ndh*C-*trn*V-UAC	50054	50277	P	4.18	1.7	2.47
nc51	A1	*ndh*C-*trn*V-UAC	50468	50658	N	2.85	1.7	1.15
nc52	B3A	*atp*E	51910	52127	P	3.85	4.85	−1
nc53	B4	*atp*E-*atp*B	52214	52383	P	1.7	4.18	−2.47
nc54	B3A	*atp*B	52500	52913	P	3.85	7.18	−3.32
nc55	B3A	*atp*B	53004	53161	P	1.7	6.02	−4.32
nc56	B3A	*atp*B	53225	53392	P	2.85	7.66	−4.81
nc57	B3A	*atp*B	53411	53739	P	1.85	8.14	−6.29
nc58	A3	*atp*B-*rbc*L	53936	54123	N	1.85	1.78	0.08
nc59	A3	*atp*B-*rbc*L	54170	54342	P	1.85	1.78	0.08
nc60	B3A	*rbc*L	55162	55356	N	1.85	10.45	−8.6
nc61	B2	*rbc*L	55814	56018	N	4.18	9.49	−5.31
nc62	A4	*rbc*L-*acc*D	56088	56453	P	3.85	1.78	2.07
nc63	A4	*rbc*L-*acc*D	56123	56306	N	1.7	1.78	−0.08
nc64	B3A	*acc*D	56690	56859	N	1.7	5.85	−4.15
nc65	B3A	*acc*D	57105	57313	N	1.85	6.56	−4.7
nc66	B3A	*acc*D	57809	57994	N	1.7	4.44	−2.74
nc67	B3A	*pet*A	61536	61883	N	3.85	5.56	−1.7
nc68	A3	*pet*A-*psb*J	62307	62606	N	3.44	5.44	−2
nc69	B3B	*psb*J	63114	63384	P	3.85	5.56	−1.7
nc70	B4	*psb*L-*psb*E	63555	64076	P	4.18	10.41	−6.24
nc71	A3	*psb*E-*pet*L-1	64296	64452	P	1.7	1.7	0
nc72	B1	*pet*L	64638	64907	N	1.7	1.85	−0.15
nc73	B2	*rpl*33	66678	66888	N	2.85	1.7	1.15
nc74	B3A	*rps*18	66932	67168	N	2.85	1.85	1
nc75	B5	*clp*P	70399	70562	P	1.7	1.7	0
nc76	B3A	*clp*P	70570	70769	P	2.85	4.66	−1.81
nc77	B3A	*psb*B	71265	71434	N	1.7	11.37	−9.66
nc78	B3A	*psb*B	71439	71905	N	5.02	12.58	−7.55
nc79	B3A	*psb*B	71946	72174	N	1.85	12.1	−10.25
nc80	B2	*psb*B	72517	72749	N	4.44	10.26	−5.82
nc81	B3A	*psb*H	73361	73511	N	2.85	3.85	−1
nc82	B5	*pet*B	73656	73853	N	5.31	6.5	−1.18
nc83	B5	*pet*D	75249	75406	N	1.7	6.31	−4.61
nc84	B3A	*rpo*A	77012	77235	P	1.85	2.85	−1
nc85	B3A	*rpo*A	77295	77501	P	2.85	4.18	−1.32
nc86	B4	*rps*11-*rpl*36	78077	78255	P	1.7	4.44	−2.74
nc87	B2	*rps*8	78685	78840	P	1.7	4.18	−2.47
nc88	B5	*rpl*16	80787	80947	P	1.7	1.7	0
nc89	B3A	*rpl*2	82845	83279	P	4.66	1.7	2.96
nc90	B3A	*rpl*2	83907	84419	P	5.02	1.7	3.32
nc91	B3A	*trn*I-CAU	84630	84965	P	1.85	1.7	0.15
nc92	B4	*ycf*15-*ndh*B	92095	93270	P	6.18	1.7	4.47
nc93	B3A	*ndh*B	93447	93677	P	3.44	1.7	1.74
nc94	B5	*ndh*B	93763	93927	P	1.7	1.7	0
nc95	B3A	*ndh*B	94061	94678	P	5.02	1.7	3.32
nc96	B3A	*rps*7	95621	95904	P	2.85	1.7	1.15
nc97	B5	*rps*12	96267	96415	P	1.7	1.7	0
nc98	B1	*rps*12	96577	97076	P	4.44	1.7	2.74
nc99	B3A	*ndh*F	110141	110353	P	2.85	1.85	1
nc100	B1	*rpl*32	110951	111234	N	1.85	1.7	0.15
nc101	B3B	*trn*L-UAG	111376	111832	N	5.31	1.7	3.61
nc102	B3A	*ccs*A	112632	112844	N	1.85	1.85	0
nc103	B3A	*ndh*D	113867	114011	P	1.7	6.56	−4.85
nc104	B3A	*ndh*I	116816	116987	P	1.85	5.18	−3.32
nc105	B5	*ndh*A	118049	118354	P	3.85	3.44	0.42
nc106	B5	*ndh*A	118405	118556	P	1.7	3.44	−1.74
nc107	B3A	*ndh*A	118767	118932	P	1.7	6.66	−4.96
nc108	B3A	*ndh*A	119109	119245	P	1.7	5.56	−3.85
nc109	B3A	*ycf*1	122874	123076	P	1.85	1.7	0.15
nc110	B3A	*ycf*1	125963	126185	P	4.66	1.7	2.96
nc111	B3A	*ycf*1	126388	126722	P	4.18	1.7	2.47
nc112	A3	*ycf*1-*trn*N-GUU	126855	127066	P	2.85	1.7	1.15
nc113	B1	*trn*R-ACG	127767	127984	P	1.85	1.7	0.15
nc114	B3A	*rrn*5S	128072	128238	P	1.7	1.7	0
nc115	B4	*rrn*4.5S-*rrn*23S	128415	128902	P	5.02	1.7	3.32
nc116	B3A	*rrn*23S	129227	129443	P	1.85	1.7	0.15
nc117	B3A	*rrn*23S	130921	131307	P	3.85	1.7	2.15
nc118	B4	*rrn*23S-*trn*A-UGC	131390	131676	P	1.85	1.7	0.15
nc119	B4	*trn*A-UGC-*trn*I-GAU	132316	132606	P	5.44	1.7	3.74
nc120	B5	*trn*I-GAU	132771	132974	P	2.85	1.7	1.15
nc121	B1	*trn*I-GAU	133315	133511	P	1.85	1.7	0.15
nc122	B2	*rrn*16S	133763	133931	P	1.85	1.7	0.15
nc123	B3A	*rrn*16S	133980	134188	P	1.85	1.7	0.15
nc124	B3A	*rrn*16S	134232	134406	P	2.85	1.7	1.15
nc125	B3A	*rrn*16S	134640	134905	P	2.85	1.7	1.15
nc126	B3A	*ycf*15	142008	142191	P	1.7	1.7	0
nc127	B3A	*ycf*2	143031	143248	P	2.85	1.7	1.15
nc128	B3A	*ycf*2	143344	143498	P	1.7	1.7	0
nc129	B3A	*ycf*2	143716	143911	P	1.85	1.7	0.15
nc130	B3A	*ycf*2	144612	144875	P	3.85	1.7	2.15
nc131	B3A	*ycf*2	144928	145108	P	1.7	1.7	0
nc132	B3A	*ycf*2	145219	145440	P	1.85	1.7	0.15
nc133	B3A	*ycf*2	145870	146044	P	1.7	1.7	0
nc134	B3A	*ycf*2	146113	146463	P	3.44	1.7	1.74
nc135	B3A	*ycf*2	146671	146882	P	1.85	1.7	0.15
nc136	B3A	*ycf*2	147068	147240	P	1.7	1.7	0
nc137	B3A	*ycf*2	147266	147609	P	4.18	1.7	2.47
nc138	B3A	*ycf*2	148116	148261	P	1.7	1.7	0
nc139	B3A	*ycf*2	148435	148594	P	1.85	1.7	0.15

a Types of noncoding RNAs. See Figure 2 for the details.

b N: negative; P: positive.

asRNA are those located on the antisense strand of a cRNA gene and overlaps with cRNA gene regions. Depending on their location relative to the corresponding coding region of the cRNA gene, the asRNA were further divided into five types: 5′ (B1), 3′ (B2), coding (B3), span (B4) and intron (B5) asRNA (Figure 2B). B1 includes those asRNA transcripts that overlap with the region upstream of the coding region of cRNA gene but do not cover the entire coding region. B2 are those overlap with the region downstream of the coding region of cRNA but do not cover the entire coding region. B3 are those either overlap with part of the coding region (B3A), or cover the entire coding region (B3B). B4 include those span two adjacent cRNA genes. B5 include those located within the intronic region of the cRNA gene. In total, 136 ncRNA transcripts have been identified (Table 2). Their sizes range from 135 bp to 1296 bp, with a mean of 263 bp. They include 18 intergenic and 118 antisense ncRNA. The numbers of ncRNA transcripts belonging to various subtypes are: A1: 1; A2: 4; A3: 9; A4: 4; B1: 9; B2: 9; B3A: 72; B3B: 6; B4: 11; B5: 11; respectively.

To determine if these ncRNA are conserved across various plant chloroplast genomes, we compared these sequences with those from *A. thaliana*. A previous study has identified 107 ncRNA in *A. thaliana* chloroplast genome [10]. In total, 45 *S. miltiorrhiza* ncRNA were found similar to 39 *A. thaliana* ncRNA using BLASTN with an a cutoff of E value <1e-5, (Table 3). Four types of mappings were identified, which are 1∶1, 1:n, n:1 and n:n respectively. “1∶1” means that one *S. miltiorrhiza* ncRNA was mapped to one and only one *A. thaliana* ncRNA. “1:n” means that one *S. miltiorrhiza* ncRNA was mapped to multiple *A. thaliana* ncRNA. “n:1” and “n;n” mean that multiple *S. miltiorrhiza* ncRNAs were mapped to one or multiple *A. thaliana* ncRNAs respectively. In particular, there are five n:1 mappings between *S. miltiorrhiza* and *A. thaliana* ncRNA. Moreover, one *S. miltiorrhiza* ncRNA (nc99) was similar to two *A. thaliana* ncRNA (*ndh*F-5′-1, *ndh*F-5′-2), a 1:n mapping. Last, nc92 and nc93 of *S. miltiorrhiza* are similar to *trnL-ndh*Bint and *ndh*B-3′ of *A. thaliana*, a n:n mapping. However, we can not exclude that possibilities that some of these n:1,1:n and n:n mappings resulted from partial transcripts. These conserved ncRNA between *S. miltiorrhiza* and *A. thaliana* may have similar functions. Further investigations are required to test this possibility.

**Table 3:** **List of ncRNAs from** ***S. miltiorrhiza*** **and** ***A. thaliana*** **sharing sequence similarity.**

*S. miltiorrhiza*	*A. thaliana*		Identity (%)	E value
ncRNAs_ID	asRNA Name	asRNA Location		
nc5	*trn*Q	6521_6646	100	3.00e-11
nc6	*psb*K	6882_7462	88.7	6.00e-22
nc7	*trn*S.1	7817_7984	97.5	1.00e-15
nc29	*trn*G-3′	9244_9484	96.3	7.00e-08
nc8	*atp*A	10342_10536	84.29	1.00e-09
nc22	*trn*D	29627_29825	91.89	6.00e-09
nc24	*psb*D	33106_33456	96.74	5.00e-42
nc25	*psb*D	33106_33456	94.44	2.00e-69
nc30	*rps14-psa*Bint	37250_37327	93.75	2.00e-08
nc35	*Ycf*3-3′	42542_42629	88.64	3.00e-08
nc41	*Rps*4-5′	45747_45929	94.44	7.00e-11
nc67	*pet*A	62045_62271	89.43	7.00e-58
nc69	*psb*J	63479_63648	90.62	2.00e-51
nc70	*psb*L-*psb*F	63805_64018	93.59	3.00e-49
nc72	orf31	65708_65805	90.36	2.00e-24
nc74	*Rps*18	67784_68222	95.36	2.00e-109
nc80	*psb*B	73677_73837	91.3	8.00e-57
nc81	*psb*H	74601_74726	96.83	3.00e-27
nc84	*rpo*A	78320_78768	84.09	2.00e-32
nc85	*rpo*A	78320_78768	86.02	4.00e-43
nc86	*Rpl*36	79502_79858	94.29	2.00e-10
nc91	*trn*I.1	86203_86389	94.65	2.00e-80
nc137	*Ycf*2.1-2	87948_88212	94.59	8.00e-98
nc136	*Ycf*2.1-3	88451_88564	94.81	1.00e-30
nc134	*Ycf*2.1-4	88960_89328	93.08	1.00e-62
nc131	*Ycf*2.1-5	89445_90769	94.94	5.00e-79
nc132	*Ycf*2.1-5	89445_90769	95.95	3.00e-105
nc133	*Ycf*2.1-5	89445_90769	87.12	4.00e-33
nc134	*Ycf*2.1-5	89445_90769	89.02	3.00e-20
nc129	*Ycf*2.1-7	91703_92005	90.73	1.00e-73
nc127	*Ycf*2.1-8	92309_92432	95.83	4.00e-18
nc92	*trnL-ndh*Bint	94377_94967	86.43	4.00e-90
nc92	*trnL-ndh*Bint	94377_94967	89.25	4.00e-25
nc92	*trnL-ndh*Bint	94377_94967	93.55	2.00e-18
nc92	*ndh*B-3′	95111_95707	95.24	3.00e-44
nc93	*ndh*B-3′	95111_95707	96.1	1.00e-110
nc95	*ndh*Bi	95910_96164	94.9	1.00e-67
nc98	*Rps*12-5′	98718_98822	98.1	1.00e-51
nc114	*rrn*4.5-*rrn*5int	107905_107963	97.83	2.00e-19
nc112	*Ycf*1.1-1	109354_109553	97.96	4.00e-21
nc110	*Ycf*1.1-2	109923_110387	94.62	9.00e-38
nc110	*Ycf*1.1-2	109923_110387	92	2.00e-14
nc99	*ndh*F-5′-1	112345_112435	85.92	1.00e-12
nc99	*ndh*F-5′-2	112475_112665	88.76	2.00e-23
nc103	*ndh*D-2	116137_116437	91.18	2.00e-07
nc105	*ndh*Ai	120852_120948	86.67	1.00e-06
nc107	*ndh*A-5′	121398_121822	92.19	7.00e-47
nc108	*ndh*A-5′	121398_121822	87.72	1.00e-11
nc110	*Ycf*1.2-7	128220_129004	94.62	9.00e-38
nc110	*Ycf*1.2-7	128220_129004	92	2.00e-14
nc111	*ycf*1.2-7	128220_129004	95.73	9.00e-110

### Validation of RNA abundance and differential expression from RNA-Seq data using ss-qPCR

We first calculated the correlation between the RNA abundance obtained from RNA-Seq and the three ss-qPCR results respectively. For these 24 pairs of ncRNA and cRNA, pearson correlation coefficients of 0.749, 0.764 and 0.779 were obtained (data not shown).

**Figure 3:**
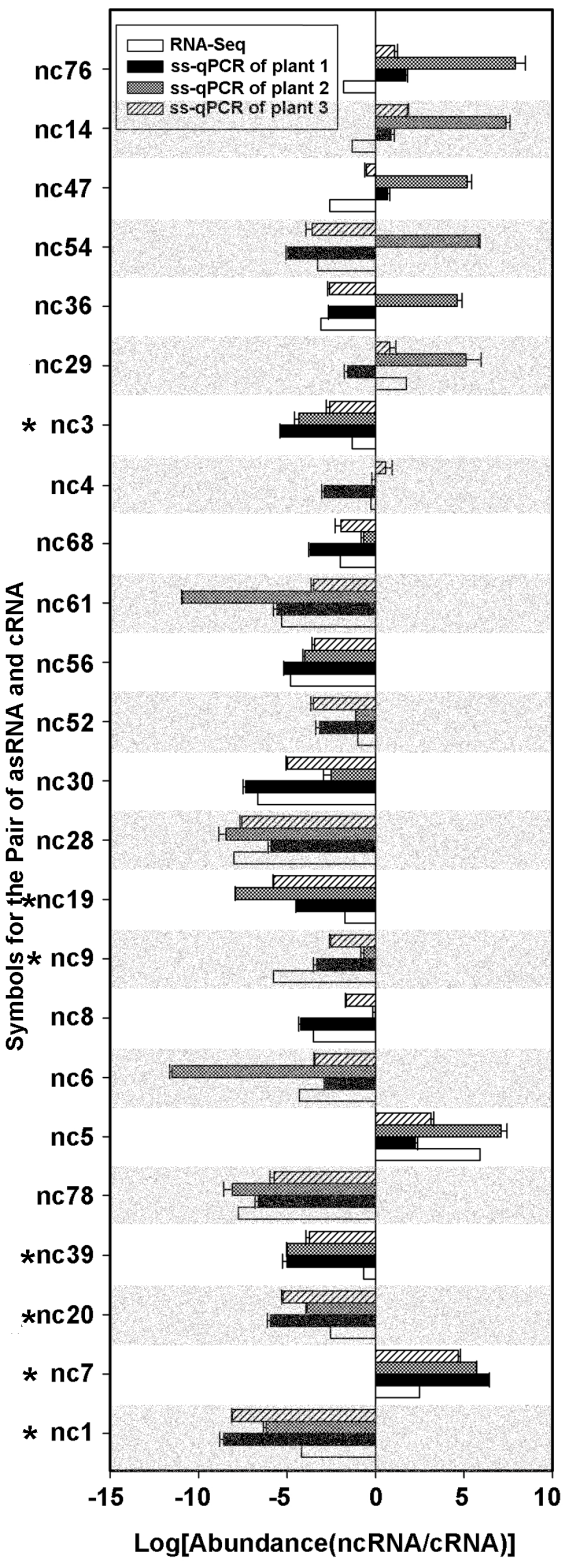
Validation of RNA-Seq data using ss-qPCR. Specific primers were designed and used to measure the abundance of the transcripts that correspond to the positive and negative strands using ss-qPCR. The log ratio (X axis) of the abundance of ncRNA and cRNA transcripts was used to measure the differential expression of this locus. (*) indicates those transcripts whose log ratio derived from the RNA-Seq result is significant different from those of ss-qPCR (*p*<0.05).

The ncRNA and its corresponding cRNA can be considered as two different conditions for differential gene expression study. We compared the log fold changes, also called log ratios, of the expression levels for ncRNA and cRNA obtained from RNA-Seq and ss-qPCR experiments. The calculation of the log ratio of ncRNA/cRNA from RNA-Seq and each of the ss-qPCR experiments is described in the method section. The log ratios are shown in Figure 3. For a pair of ncRNA and cRNA, the statistical significance between the RNA-Seq and the ss-qPCR results were tested using one-sample *t* test (Table S3 in File S1). A *p* value <0.05 was considered significantly different. In total, 17 out of 24 pairs of ncRNA and cRNA (70%) did not exhibit significantly different gene expression between RNA-Seq and ss-qPCR results (Figure 3). The 7 pairs showed significantly different expression between the RNA-Seq and ss-qPCR results are indicated with “*”. Overall, these findings demonstrate the reasonably good quality of our RNA-Seq data.

### Interplay between asRNA and cRNA expression

**Figure 4:**
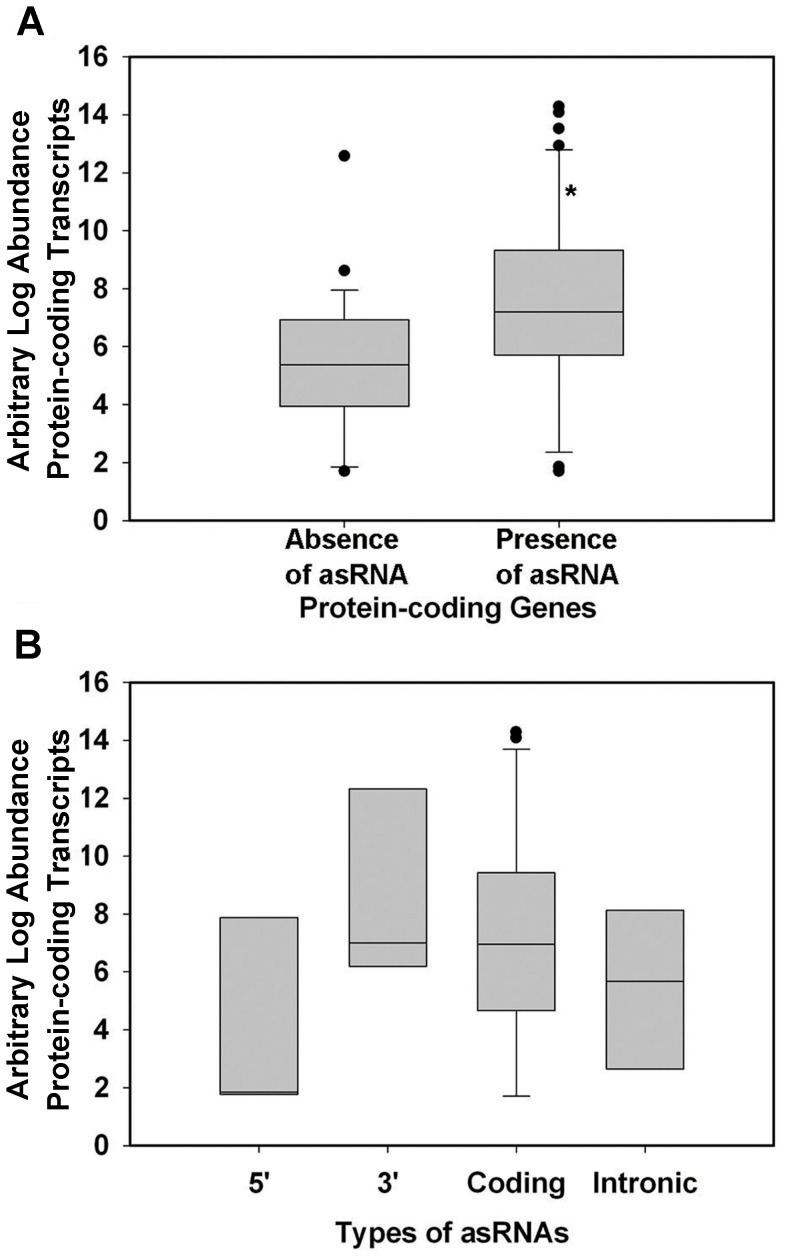
Effects of asRNA on the expression of the corresponding cRNA. (A) log abundance of cRNA with and without asRNA. (B) log abundance of cRNA in the presence of four different types of asRNA. The line in the middle of the box represents the mean. The upper and lower borders of the boxes indicate the 75% and 25% quantile lines, respectively. The ends of the whiskers represent two standard deviations from the mean. “*” indicates that the difference is significant (*p*<0.05) based on a Student's *t*-test.

To detect any interplay between cRNA,and asRNA, we determined the expression levels of cRNA and asRNA transcripts of all 80 protein-coding genes (Table 2). It should be pointed out that, in Table 1, the start and end positions of cRNA are defined based on those of ncRNA. While in Table 1, the start and end positions of cRNA were defined based on those from gene annotations. At a result, the expression levels are slightly different for the same cRNA in Table 1 and 2. The expression of antisense strands was not detected in 27 of the 80 protein coding genes. A Students' *t*-test showed that the abundance of cRNA with asRNA expression was significantly higher than those of cRNA with no ncRNA expression (*p*<0.05) (Figure 4A). This result suggests that asRNA expression promotes cRNA expression on the opposite strand (see the Discussion section for additional details). As previously described, asRNA can be divided into four types based on their relative positions to the coding transcripts. To detect any correlation between the asRNA locations and cRNA expression, we plotted the asRNA expression by their types (Figure 4B). The average abundance of cRNA with 3′ coding asRNA and intronic asRNA were similar to those shown in Figure 4A. However, the abundance of cRNA with 5′ asRNA was considerably lower but the difference was not statistically significant, which indicates the possible repressive effects of asRNA on cRNA abundance.

### Identification of DNA modification sites and DNA modification motifs

**Figure 5:**
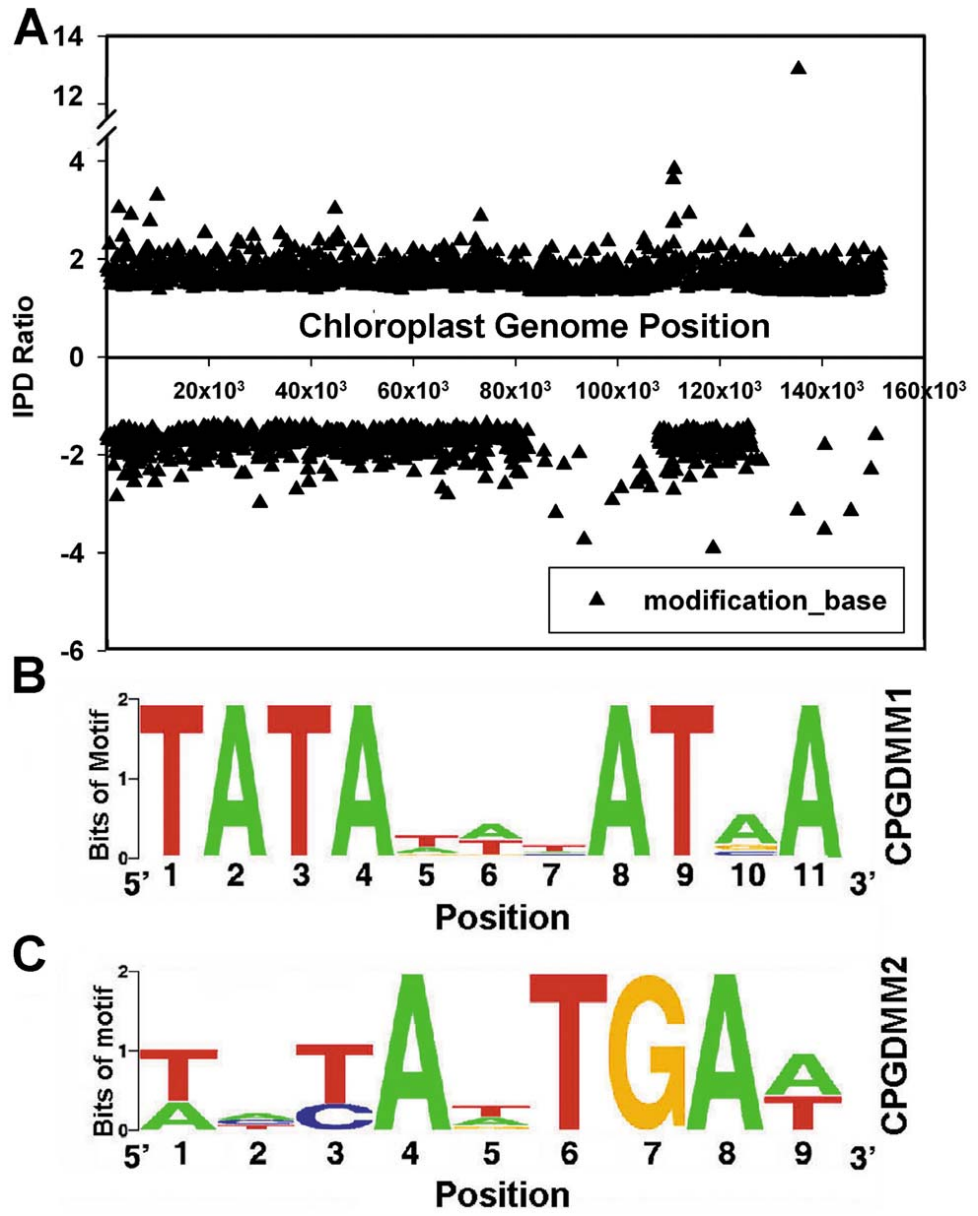
Identification of putative DNA modification sites and motifs in the *S. miltiorrhiza* chloroplast genome using SMRT technology. (A) Distribution of interpulse duration (IPD) Ratio across the genome. “▴” indicate that the corresponding IPD ratio (“▴”) has a *p*<0.05, which suggests that the base at this position is modified. (B) Sequence LOGO for putative DNA modification motif 1 (DMM1). (C) Sequence LOGO for putative DNA modification motif 2 (DMM2).

DNA modifications influence cellular functions. SMRT technology no only produces considerably longer reads than other second-generation DNA sequencing technologies but also provides detailed kinetic information of the DNA polymerase. As described earlier, SMRT sequencing technology detects the fluorescent signal during the period when the DNA polymerase is “holding” a nucleotide. These form fluorescent “pulses” as DNA polymerase continue incorporating nucleotides into the elongating DNA strand. The interpulse duration (IPD) is the time difference between two “pulses”. IPD ratio is calculated as [IPD at a site at a condition under study]/[IPD at the same site at the control condition], The IPD changes significantly in the presence of DNA modifications. Based on the IPD ratio, the DNA modification status of the base can be inferred [20]. Using the DNA modification analysis module of SMRT Portal 1.3.2, we identified 2687 (*p*<0.01) putatively modified bases throughout the genome (Figure 5A). Among these, 90.3% IPD ratio ranged from -2 to 2. We identified two DNA motifs using the motif analysis module, namely, “TATANNNATNA” (Figure 5B) and “WNYANTGAW” (Figure 5C), whose percentage of putatively modified sites was 35/97 (36.1%) and 91/369 (24.7%), respectively (Table 4). Detailed information on the sites belonging to motifs 1 and 2 is included in Tables S4 and S5 in File S1, respectively.

**Table 4:** **Putative DNA modification motifs associated with base modifications.**

DMM id	Motif Sequence	Total No. of Motifs in Genome	No. of Motifs Modified	Fraction of Motifs Modified	Mean IPD Ratio	Mean Coverage	Position Modified
CPGDMM1	TATANNNATNA	97	35	0.36	1.71	66.00	−1
CPGDMM2	WNYANTGAW	369	91	0.25	1.77	84.71	−3

### Interplay between DNA modification and gene expression

**Figure 6:**
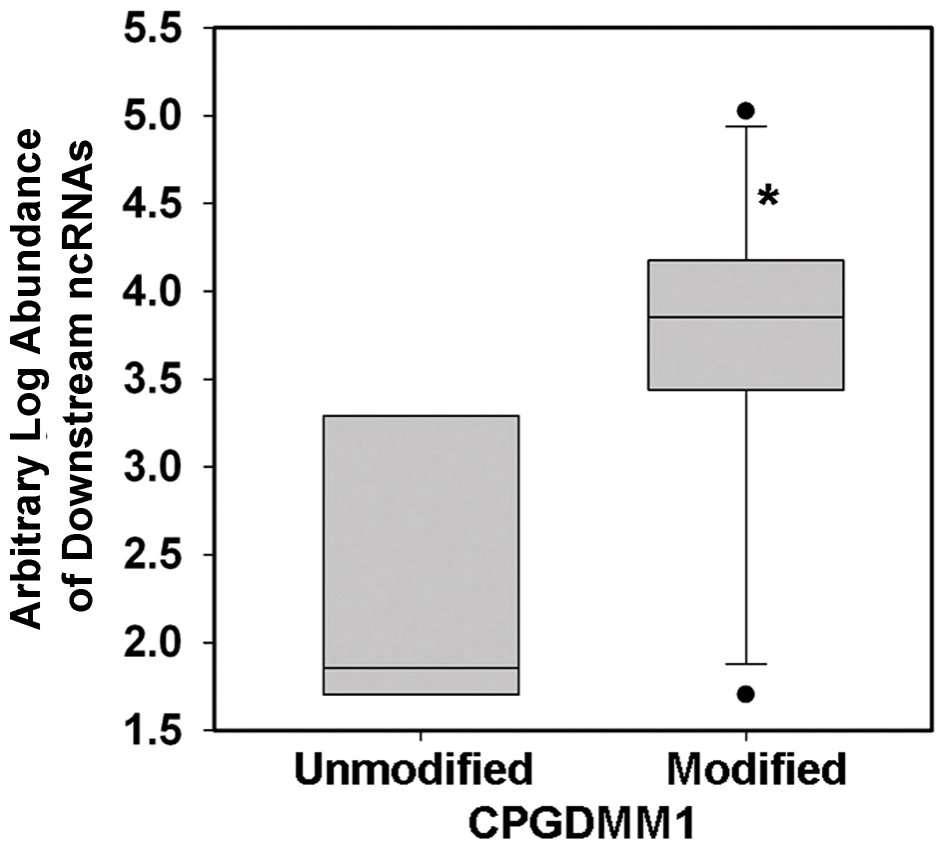
Effects of modification at the CPGDMM1 motif on the abundance of ncRNA. Box plot showing the differences in the expression of two groups of ncRNA transcripts (x axis), which are defined based on whether or not their upstream CPGDMM1 motifs are significantly modified. The *y* axis shows the arbitrary expression levels of ncRNA transcripts in these two groups. The line in the middle of the box represents the mean. The upper and lower borders of the boxes indicate the 75% and 25% quantile lines, respectively. The ends of the whiskers represent two standard deviations from the mean. “*” indicate that the difference is statistically significant using ANOVA (*p*<0.01).

The sequence of DMM1, “TATANNNATNA,” is similar to that of a TATA box frequently found in the core promoter of prokaryotes. Therefore, whether the modifications of these putative TATA box motifs would affect the expression of genes downstream of the modification sites is of significant interest. We extracted the transcripts corresponding to genes coding for protein, tRNA and ncRNA downstream of these DMM1 motifs. The downstream transcripts were further classified into three groups based on the distance between the modification motifs and the start of the downstream genes. For the group in which all transcripts were ncRNAs and the distance between DMM1 and the start of ncRNAs ranged from 100 bp to 500 bp (Table S6 in File S1), the presence of DNA modification was significantly correlated with higher ncRNA expression levels. The difference was significant with *p*<0.01 (Figure 6 and Table S7 in File S1).

### Prediction of sites for foreign gene insertion

**Figure 7:**
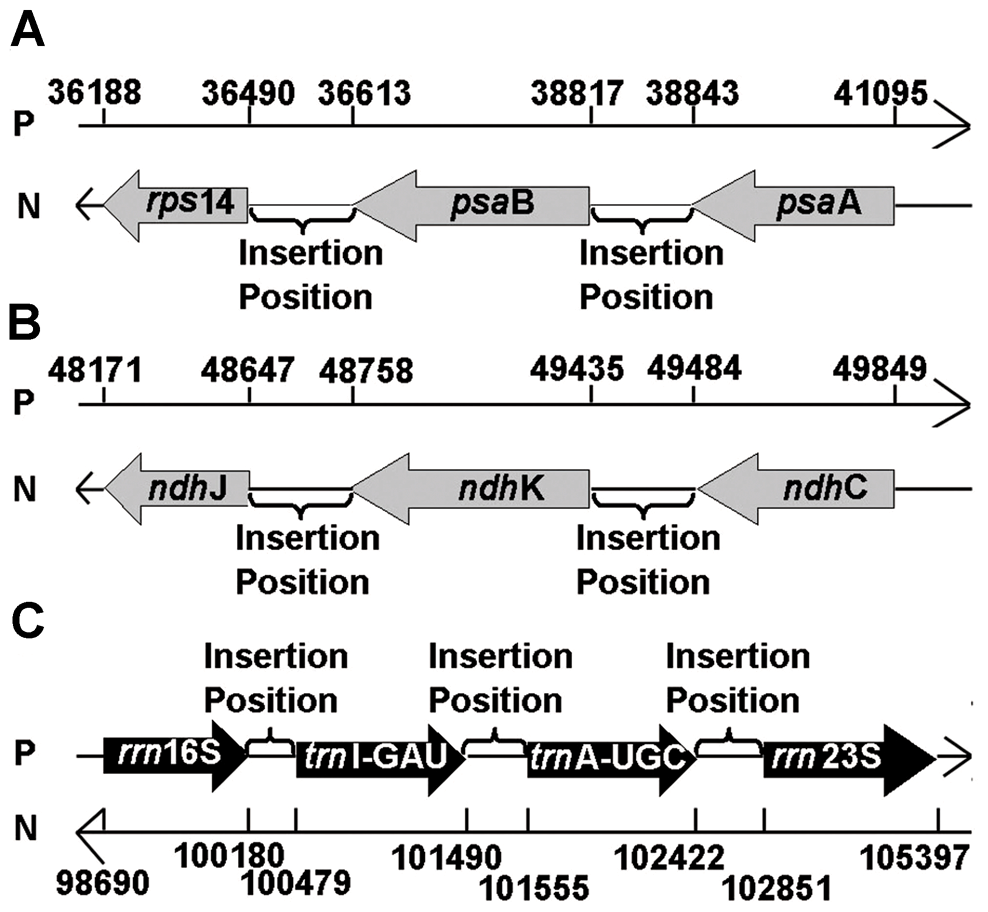
Schematic representation of the optimal insertion positions for foreign genes identified in the current study. The names, genomic positions, and orientations of the genes in three different loci, namely, *rps*14-*ps*aB-*psa*A, *ndh*J-*ndh*K-*ndh*C, and *rrn*16S-(*trn*I-GAU)-(*trn*A-UGC)-*rrn*23S, are shown in panels (A), (B), and (C), respectively. P: positive strand; N: negative strand.

We systematically analyzed the regions to select those that are suitable insertion sites. Our criteria included the following: (1) the region should have minimal effects on the expression of endogenous chloroplast genes; and (2) it has potential to be highly expressed. In particular, we selected locations that are in the intergenic regions of polycistronic sites and at which the relative expression levels of genes are high. The top-ranking regions are presented in Figure 7. Interestingly, these regions include *trn*I-GAU and *trn*A-UGC (Figure 7C), which have been widely used in chloroplast-based genetic engineering [34]. The consistency between our prediction and those already used experimentally indicates that our selection criteria are reasonably well.

## Discussion

### RNA-Seq technology

The current study takes advantage of two advanced technologies, RNA-Seq and SMRT, to identify asRNA and DNA modifications and then characterize their effects on gene expression in the *S. miltiorrihiza* chloroplast genome. RNA-Seq is a revolutionary tool for transcriptomic analysis [35]. It has several advantages over other transcriptomic methods such as tiling microarray and cDNA or EST sequencing [36], namely, independence from genomic sequence, low background noise, wide dynamic range to quantify gene expression level, and high throughput, among others. Moreover, strand-specific RNA-Seq methods provide information on the orientation of transcripts, which is valuable for transcriptome annotation particularly for regions with overlapping transcription from opposite directions [25]. As demonstrated in the current study, strand-specific RNA-Seq technology enables the detection of polycistrons and asRNA from chloroplast transcriptome, which is essential for interpreting the functional elements of chloroplast genomes.

### SMRT technology

SMRT is a novel technology for detecting DNA methylation while simultaneously determining the context of the corresponding DNA sequence [20]. Before the development of SMRT technology, the most commonly used methods for detecting DNA modification are largely limited to bulk methods such as chromatography, mass spectroscopy, electrochemistry, radioactive labeling, immunochemical assays, and sensitivity to restriction enzymes [20]. However, these approaches suffer from low resolution and an inability to identify the precise sequence context of the methylation site(s). The SMRT technology allows the collection of kinetic data for the enzyme during the incorporation of each dNTP into the DNA strand. Significant changes in kinetic parameters such as IPD ratio should be observed when the DNA polymerase encounters m6A, m5C, or 5-hmC on the template strand. These distinct kinetic signatures allow the identification of the type and position of the base modification in the DNA template [20]. The SMRT technology is likely to enhance DNA modification studies on samples that were not previously accessible for this type of research, such as organelle genomes.

### Gene expression regulation by ncRNA

Several mechanisms have been proposed to explain the regulatory role of ncRNA, including transcription itself (transcriptional interference), as well as the formation of duplexes of RNA–DNA (chromatin remodeling) and RNA–RNA (dsRNA-dependent RNA formation) [37]. Under the first model, the abundance of natural sense-antisense transcript (SAT) pairs was found to be inversely correlated and antisense transcription leads to the downregulation of the corresponding sense transcript. Under the second model, the antisense transcript recruits histone-modifying enzymes that alter the chromatin structure, resulting in sense-transcript repression. Consistently, sense transcription is induced in the absence of antisense transcripts. Under the last model, double-stranded RNA formation spans an intron, which inhibits splicing and leads to a mature transcript with a retained intron.

Although the majority of studies on asRNA focused on those from nuclear genomes, some studies focused on asRNA in the chloroplast genome. For example, overexpression of a natural chloroplast-encoded antisense RNA (AS5) in tobacco destabilizes 5S rRNA and retards plant growth, possibly because AS5 prevents the accumulation of misprocessed 5S rRNA and controls its stoichiometry [18]. The majority of previous studies showed that asRNA expression is negatively correlated with that of the cRNA. However, positive correlation has also been found between asRNA and cRNA in studies on human breast epithelium and mammals [38], [39].

In our study, we showed that at the genome scale, the cRNA expression levels in SATs are significantly higher than those not in SAT (*p*<0.05) (Figure 4A). In other words, cRNA expression is positively correlated with asRNA expression. One likely mechanism is that asRNA expression might open up the chromatin and increase the accessibility of the corresponding region to the transcriptional machinery for cRNA, causing higher expression of the corresponding cRNA. Nevertheless, we cannot exclude the possibility that cRNA expression causes the higher asRNA expression in the same manner. Additional studies are in need to test these hypotheses.

### Gene expression regulation by DNA modification

Previous studies indicated that gene transcription and DNA methylation are closely interwoven processes [40]. In mammalian genomes, DNA methylation is generally associated with silent CpG island promoters. However, the majority of CpG island promoters remain methyl-free regardless of their expression status [41]. In human testicular cancer, the addition of methylation inhibitors leads to downregulation of ncRNA expression [42]. In plants, a spontaneous mutation causing a single nucleotide polymorphism (SNP) between wild-type and mutant genes alter the secondary structure of LDMAR, an lncRNA gene. This change increases methylation in the putative promoter region of LDMAR, which reduces LDMAR transcription specifically under long-day conditions [43].

Although the expression regulation of chloroplast genes has been intensively studied [10], the presence of ncRNA and DNA modifications and their interaction with the expression of cRNA remain unclear. In the current study, we use SMRT technology and detected possible base modifications in chloroplast genomes. Furthermore, we identified two motifs that are associated with base modifications. As the first motif is similar to the TATA box in sequence, we hypothesize that it might regulate the expression of downstream genes as a transcriptional factor binding site. After classifying the downstream genes by gene type and their distance to the putative TATA box, the modification was found to be associated with significantly higher expression of ncRNA but not those protein-coding and tRNA genes. One explanation is that proteins and tRNA-coding genes might be regulated by specific elements such as promoters and enhancers, and that base modification plays a limited role in their expression regulation. However, ncRNA expression regulation is less specific, and the modification of its upstream TATA box–like motif leads to significant differences in expression. Contrary to the general thoughts that DNA methylation downregulate gene expression, we found that CPGDMM1 methylation is correlated with increased ncRNA expression. These observations may reflect the unique mechanisms present in chloroplast genomes. Further investigation is needed to confirm these associations and to elucidate possible mechanisms.

### Chloroplast based genetic engineering

Chloroplast vector systems have several advantages and consequently have multiple biotechnological applications [4]. One of our rationales for this study is to lay the foundation for designing efficient, chloroplast-based transformation vectors for *S. miltiorrhiza*. Chloroplast genomes are most abundant in the leaves. Therefore, focusing on the chloroplast genome characteristics in the leaves is reasonable. After obtaining a detailed expression map of *S. miltiorrhiza* chloroplasts in the leaves, we systematically predict regions that are potential insertion sites for foreign genes. Two of them have already been commonly used as sites for foreign gene insertion. Furthermore, the region between *psa*A and *psa*B on polycistron pc8 and that between *ndhK* and *ndhC* on polycistron pc9 (Figure 7) were also identified as the most preferred insertion sites. These two sites have not been used previously and represent novel insertion site for foreign genes. An extensive validation of these sites for their application in chloroplast-based genetic engineering research will be the subject of a separate study.

### Technical limitations

Second- and third-generation DNA sequencing technologies have made feasible the detection of ncRNA and DNA modifications at the genome scale. However, formidable experimental difficulties are inherent to antisense RNA and DNA modification research. First, most asRNA transcripts are expressed at significantly low levels and are thus difficult to be validated using classical technologies such as northern blot analysis and in situ hybridization. Second, the intricate relationship between sense and antisense transcripts means that experimental perturbation of one transcript inevitably interferes with the expression of the other transcript. Consequently, determining the biological functions of the transcripts by knocking-in and knocking-out the transcript alone is complicated. Third, while the SMRT technology has been shown to be able to detect potential DNA modifications, validating these modifications remains a challenging task. Forth, validation of the presence and function of asRNA and DNA modifications in the chloroplast is even more difficult. Any perturbation experiment has to consider how to specifically target the transcript in the organelle because the molecule has to be delivered to the chloroplast. Furthermore, excluding interference from the nuclear transcripts is difficult because the interaction between those from the nucleus and those from the chloroplast is well known.

In summary, validating the discovery described in this study is technically formidable at present time. Nevertheless, the data presented in this study have demonstrated the complexity of gene expression regulation by asRNA and DNA modification.

## Supporting Information

File S1 **Contains the files: Figure S1.** Characteristics of SMRT sequencing results: (A) length distribution of sequence reads; (B) depth of coverage across the assembled chloroplast genome; and (C) abundance of various lengths of reads mapped to the chloroplast genome. **Table S1.** Primers used for sequence assembly validation. **Table S2.** Primer sets used for strand-specific real-time qPCR. **Table S3.** Statistical testing of RNA-Seq and ss-qPCR results using one-sample *t* test. **Table S4.** Detailed information on motif CPGDMM1. **Table S5.** Detailed information on motif CPGDMM2. **Table S6.** Associations between DNA modification site (DMS) and downstream ncRNA expression. **Table S7.** Analysis of the effect of DNA modification at the CPGDMM1 motif on the abundance of ncRNA using ANOVA.
(DOC)
