# Supplementary material for: Complex Interplay among DNA Modification, Noncoding RNA Expression and Protein-Coding RNA Expression in Salvia miltiorrhiza Chloroplast Genome
Source: PLoS One. 2014 Jun 10;9(6):e99314. doi: 10.1371/journal.pone.0099314 (PMC4051680; doi:10.1371/journal.pone.0099314)

**Supporting Tables**

**Table S1 . Primers used for sequence assembly validation.**

| **Primer ID** | **Location** | **Primer sequence(5′–3′)** | **Primer length (bp)** | **Product size (bp)** |
| --- | --- | --- | --- | --- |
| Cpg_b1-F | LSC/IRa | CCTAAAGCGCGTACTTCTGTTT | 22 | 758 |
| Cpg_b1-R |  | AAAATTACCTTCTGGGGAGGTC | 22 |  |
| Cpg_b2-F | IRa/SSC | AGAAACAGCTTCCGAAATGAAG | 22 | 756 |
| Cpg_b2-R |  | AACCAAATGGTTAGCACCTTCT | 22 |  |
| Cpg_b3-F | SSC/IRb | CACAATCGAGTTCTTGTTTCCA | 22 | 748 |
| Cpg_b3-R |  | ATTTAGGCAGAATACCGTCACC | 22 |  |
| Cpg_b4-F | IRb/LSC | CGAACCAGAAAAGTTTGGGTAG | 22 | 746 |
| Cpg_b4-R |  | GAAGCTCCAACAAATGGCTAAG | 22 |  |

**Table S2. Primer sets used for strand-specific real-time qPCR**

| **AsRNA ID** | **Corresponding coding genes** | **asRNA Strand** | **Target Template** | **Primer ID** | **Primer Sequence** | | |
| --- | --- | --- | --- | --- | --- | --- | --- |
|  |  |  |  |  | **Reverse Transcription** | **ss-qPCR** | |
| nc1 | *psb*A | P | asRNA | Cpg_1_asRNA | CCAGATCGTTCGAGTCGTGCTTAGTTTCCGTTTGGGTATG | mRNAtag | CCAGATCGTTCGAGTCGT (+) |
|  |  |  |  |  |  | Cpg_1_F | GGCCTATTGGGTAGATCAAGAA |
|  |  |  | cRNA | Cpg_1_cRNA | GCTAGCTTCAGCTAGGCATCGGCCTATTGGGTAGATCAAGAA | cRNAtag | GCTAGCTTCAGCTAGGCATC |
|  |  |  |  |  |  | Cpg_1_R | GCTTAGTTTCCGTTTGGGTATG |
| nc7 | *trn*S-GCU | P | asRNA | Cpg_2_asRNA | GCTAGCTTCAGCTAGGCATCCCGGGTCGTGAGAATAAGAA | cRNAtag |  |
|  |  |  |  |  |  | Cpg_2_F | CGCCAACACTCGAACAAA |
|  |  |  | cRNA | Cpg_2_cRNA | CCAGATCGTTCGAGTCGTCGCCAACACTCGAACAAA | mRNAtag |  |
|  |  |  |  |  |  | Cpg_2_R | CCGGGTCGTGAGAATAAGAA |
| nc20 | *pet*N-*psb*M | N | asRNA | Cpg_7_asRNA | GCTAGCTTCAGCTAGGCATCTCGAGATCGTGGTTGTTTAAGG | cRNAtag |  |
|  |  |  |  |  |  | Cpg_7_R | AAGCCTAAACCGCCTTTCTAA |
|  |  |  | cRNA | Cpg_7_cRNA | CCAGATCGTTCGAGTCGTAAGCCTAAACCGCCTTTCTAA | mRNAtag |  |
|  |  |  |  |  |  | Cpg_7_F | TCGAGATCGTGGTTGTTTAAGG |
| nc39 | *ycf*3-*trn*S-GGA | P | asRNA | Cpg_9_asRNA | GCTAGCTTCAGCTAGGCATCGGCTTTAGAGTTCTCAACAACAAA | cRNAtag |  |
|  |  |  |  |  |  | Cpg_9_F | TACATAGCAGGTATACAATCCACTATAC |
|  |  |  | cRNA | Cpg_9_cRNA | CCAGATCGTTCGAGTCGTTACATAGCAGGTATACAATCCACTATAC | mRNAtag |  |
|  |  |  |  |  |  | Cpg_9_R | GGCTTTAGAGTTCTCAACAACAAA |
| nc3 | *trn*K-UUU | P | asRNA | Cpg_10_asRNA | CCAGATCGTTCGAGTCGTGAGAGGGTAGAGAATCTGTTGATAAG | mRNAtag |  |
|  |  |  |  |  |  | Cpg_10_F | TGATACATAGTGCGATACAGTCAAA |
|  |  |  | cRNA | Cpg_10_cRNA | GCTAGCTTCAGCTAGGCATCTGATACATAGTGCGATACAGTCAAA | cRNAtag |  |
|  |  |  |  |  |  | Cpg_10_R | GAGAGGGTAGAGAATCTGTTGATAAG |
| nc4 | *trn*K-UUU | P | AsRNA | Cpg_11_asRNA | CCAGATCGTTCGAGTCGTGGGTTGCTAACTCAATGGTAGA | mRNAtag |  |
|  |  |  |  |  |  | Cpg_11_F | GTCAGTCGCGGTCTTCAAA |
|  |  |  | cRNA | Cpg_11_cRNA | GCTAGCTTCAGCTAGGCATCGTCAGTCGCGGTCTTCAAA | cRNAtag |  |
|  |  |  |  |  |  | Cpg_11_R | GGGTTGCTAACTCAATGGTAGA |
| nc5 | *trn*Q-UUG | P | AsRNA | Cpg_12_asRNA | CCAGATCGTTCGAGTCGTCTGGAGTTGACAAACAACCAATAA | mRNAtag |  |
|  |  |  |  |  |  | Cpg_12_F | AAACCCGTTGCCTTACCA |
|  |  |  | cRNA | Cpg_12_cRNA | GCTAGCTTCAGCTAGGCATCAAACCCGTTGCCTTACCA | cRNAtag |  |
|  |  |  |  |  |  | Cpg_12_R | CTGGAGTTGACAAACAACCAATAA |
| nc6 | *psb*K-*psb*I | N | AsRNA | Cpg_13_asRNA | GCTAGCTTCAGCTAGGCATCTAGTCGGCATAAATCAGGCTTAC | cRNAtag |  |
|  |  |  |  |  |  | Cpg_13_R | GGTTAGGGTCTTTGACTGGATG |
|  |  |  | cRNA | Cpg_13_cRNA | CCAGATCGTTCGAGTCGTGGTTAGGGTCTTTGACTGGATG | mRNAtag |  |
|  |  |  |  |  |  | Cpg_13_F | TAGTCGGCATAAATCAGGCTTAC |
| nc8 | *atp*A | P | AsRNA | Cpg_14_asRNA | CCAGATCGTTCGAGTCGTCTGCCATTAACGTGGGTATCT | mRNAtag |  |
|  |  |  |  |  |  | Cpg_14_F | ACTTACCGGCTACTTGTTTCAT |
|  |  |  | cRNA | Cpg_14_cRNA | GCTAGCTTCAGCTAGGCATCACTTACCGGCTACTTGTTTCAT | cRNAtag |  |
|  |  |  |  |  |  | Cpg_14_R | CTGCCATTAACGTGGGTATCT |
| nc9 | *atp*A | P | AsRNA | Cpg_15_asRNA | CCAGATCGTTCGAGTCGTCTCCGTATACGAACCTCTTCAG | mRNAtag |  |
|  |  |  |  |  |  | Cpg_15_F | CGGTCTGTCTGTCTCCAATAA |
|  |  |  | cRNA | Cpg_15_cRNA | GCTAGCTTCAGCTAGGCATCCGGTCTGTCTGTCTCCAATAA | cRNAtag |  |
|  |  |  |  |  |  | Cpg_15_R | CTCCGTATACGAACCTCTTCAG |
| nc14 | *rpo*C2 | P | AsRNA | Cpg_16_asRNA | CCAGATCGTTCGAGTCGTTTCCGAAGAGGGAATGTCTAATG | mRNAtag |  |
|  |  |  |  |  |  | Cpg_16_F | CCAATAAGAGGGCTCGGTAAC |
|  |  |  | cRNA | Cpg_16_cRNA | GCTAGCTTCAGCTAGGCATCCCAATAAGAGGGCTCGGTAAC | cRNAtag |  |
|  |  |  |  |  |  | Cpg_16_R | TTCCGAAGAGGGAATGTCTAATG |
| nc19 | *pet*N | N | AsRNA | Cpg_17_asRNA | GCTAGCTTCAGCTAGGCATCGCAACGCACTTTGGACTATTT | cRNAtag |  |
|  |  |  |  |  |  | Cpg_17_R | CGAAGTACAAAGACGGGAGAA |
|  |  |  | cRNA | Cpg_17_cRNA | CCAGATCGTTCGAGTCGTCGAAGTACAAAGACGGGAGAA | mRNAtag |  |
|  |  |  |  |  |  | Cpg_17_F | GCAACGCACTTTGGACTATTT |
| nc28 | *psb*C | N | AsRNA | Cpg_18_asRNA | GCTAGCTTCAGCTAGGCATCACTGCTTATCCTAGCGAGTTT | cRNAtag |  |
|  |  |  |  |  |  | Cpg_18_R | CCTTGAGCCGATCCTACATTA |
|  |  |  | cRNA | Cpg_18_cRNA | CCAGATCGTTCGAGTCGTCCTTGAGCCGATCCTACATTA | mRNAtag |  |
|  |  |  |  |  |  | Cpg_18_F | ACTGCTTATCCTAGCGAGTTT |
| nc29 | *trn*G-GCC | N | AsRNA | Cpg_19_asRNA | GCTAGCTTCAGCTAGGCATCTGGCCTGATACTGCACAAATA | cRNAtag |  |
|  |  |  |  |  |  | Cpg_19_R | AATCGAACCCGCATCTTCTC |
|  |  |  | cRNA | Cpg_19_cRNA | CCAGATCGTTCGAGTCGTAATCGAACCCGCATCTTCTC | mRNAtag |  |
|  |  |  |  |  |  | Cpg_19_F | TGGCCTGATACTGCACAAATA |
| nc30 | *rps*14-*psa*B | P | AsRNA | Cpg_20_asRNA | CCAGATCGTTCGAGTCGTAGGCCTGCCTTCTTATATTTCT | mRNAtag |  |
|  |  |  |  |  |  | Cpg_20_F | CCTCTTCTTCTCCCTCTGAATC |
|  |  |  | cRNA | Cpg_20_cRNA | GCTAGCTTCAGCTAGGCATCCCTCTTCTTCTCCCTCTGAATC | cRNAtag |  |
|  |  |  |  |  |  | Cpg_20_R | AGGCCTGCCTTCTTATATTTCT |
| nc36 | *ycf*3 | P | AsRNA | Cpg_21_asRNA | CCAGATCGTTCGAGTCGTGGGCCAATAATAACTGCGTACT | mRNAtag |  |
|  |  |  |  |  |  | Cpg_21_F | CACCGCTGCTCAAGACTTT |
|  |  |  | cRNA | Cpg_21_cRNA | GCTAGCTTCAGCTAGGCATCCACCGCTGCTCAAGACTTT | cRNAtag |  |
|  |  |  |  |  |  | Cpg_21_R | GGGCCAATAATAACTGCGTACT |
| nc47 | *ndh*J | P | AsRNA | Cpg_23_asRNA | CCAGATCGTTCGAGTCGTCCCGAGGATTGGCATTCTATT | mRNAtag |  |
|  |  |  |  |  |  | Cpg_23_F | CTGGTTGATCCACACCATACTC |
|  |  |  | cRNA | Cpg_23_cRNA | GCTAGCTTCAGCTAGGCATCCTGGTTGATCCACACCATACTC | cRNAtag |  |
|  |  |  |  |  |  | Cpg_23_R | CCCGAGGATTGGCATTCTATT |
| nc52 | *atp*E | P | AsRNA | Cpg_24_asRNA | CCAGATCGTTCGAGTCGTTCCACAAGAAGCTCAGGAAAC | mRNAtag |  |
|  |  |  |  |  |  | Cpg_24_F | GGAGAGCTAGATTTGCCTCAATTA |
|  |  |  | cRNA | Cpg_24_cRNA | GCTAGCTTCAGCTAGGCATCGGAGAGCTAGATTTGCCTCAATTA | cRNAtag |  |
|  |  |  |  |  |  | Cpg_24_R | TCCACAAGAAGCTCAGGAAAC |
| nc54 | *atp*B-1 | P | AsRNA | Cpg_25_asRNA | CCAGATCGTTCGAGTCGTGGATCGTTGGTGAGGAACATTA | mRNAtag |  |
|  |  |  |  |  |  | Cpg_25_F | AATTCGTCCAACCCAAGGATAG |
|  |  |  | cRNA | Cpg_25_cRNA | GCTAGCTTCAGCTAGGCATCAATTCGTCCAACCCAAGGATAG | cRNAtag |  |
|  |  |  |  |  |  | Cpg_25_R | GGATCGTTGGTGAGGAACATTA |
| nc56 | *atp*B-3 | P | AsRNA | Cpg_26_asRNA | CCAGATCGTTCGAGTCGTCGCCTGCCTTTATACAGTTAGAT | mRNAtag |  |
|  |  |  |  |  |  | Cpg_26_F | CACGGCGATAAGGTGCTAAA |
|  |  |  | cRNA | Cpg_26_cRNA | GCTAGCTTCAGCTAGGCATCCACGGCGATAAGGTGCTAAA | cRNAtag |  |
|  |  |  |  |  |  | Cpg_26_R | CGCCTGCCTTTATACAGTTAGAT |
| nc61 | *rbc*L-2 | N | AsRNA | Cpg_27_asRNA | GCTAGCTTCAGCTAGGCATCGAGTAGCTGTAGAAGCGTGTG | cRNAtag |  |
|  |  |  |  |  |  | Cpg_27_R | CAGCTAGTTCAGGACTCCATTT |
|  |  |  | cRNA | Cpg_27_cRNA | CCAGATCGTTCGAGTCGTCAGCTAGTTCAGGACTCCATTT | mRNAtag |  |
|  |  |  |  |  |  | Cpg_27_F | GAGTAGCTGTAGAAGCGTGTG |
| nc68 | *pet*A-*psb*J | N | AsRNA | Cpg_28_asRNA | GCTAGCTTCAGCTAGGCATCCCTTGTAGCGCACTCTTAGTC | cRNAtag |  |
|  |  |  |  |  |  | Cpg_28_R | AAGGGTGGTAAAGCCCTAAAG |
|  |  |  | cRNA | Cpg_28_cRNA | CCAGATCGTTCGAGTCGTAAGGGTGGTAAAGCCCTAAAG | mRNAtag |  |
|  |  |  |  |  |  | Cpg_28_F | CCTTGTAGCGCACTCTTAGTC |
| nc76 | *clp*P-2 | P | AsRNA | Cpg_29_asRNA | CCAGATCGTTCGAGTCGTTGCATCTTGGGTTGACGTATAG | mRNAtag |  |
|  |  |  |  |  |  | Cpg_29_F | TTCTTGGGCGAAACAGAGG |
|  |  |  | cRNA | Cpg_29_cRNA | GCTAGCTTCAGCTAGGCATCTTCTTGGGCGAAACAGAGG | cRNAtag |  |
|  |  |  |  |  |  | Cpg_29_R | TGCATCTTGGGTTGACGTATAG |
| nc78 | *psb*B-2 | N | AsRNA | Cpg_30_asRNA | GCTAGCTTCAGCTAGGCATCAGGCGGAACTGTAACGAATC | cRNAtag |  |
|  |  |  |  |  |  | Cpg_30_R | CCAATAGACCCAATGCCAGATA |
|  |  |  | cRNA | Cpg_30_cRNA | CCAGATCGTTCGAGTCGTCCAATAGACCCAATGCCAGATA | mRNAtag |  |
|  |  |  |  |  |  | Cpg_30_F | AGGCGGAACTGTAACGAATC |

**Table S3 Statistical testing of RNA-Seq and ss-qPCR results using one-sample *t* test**

| **ID** | **ss-qPCR of plant 1** | **ss-qPCR of plant 2** | **ss-qPCR of plant 3** | **Mean** | **STDEV** | **t_value** | **RNA-Seq** | **Degree of freedom** | **t_probability** | **Is significant?** |
| --- | --- | --- | --- | --- | --- | --- | --- | --- | --- | --- |
| nc1 | -8.5836649 | -6.1831684 | -8.0891294 | -7.6186543 | 1.2675196 | -4.6715512 | -4.2 | 2 | 0.97855 | yes |
| nc7 | 6.3800786 | 5.6956997 | 4.6771564 | 5.5843116 | 0.8569081 | 6.2746816 | 2.48 | 2 | 0.012235 | yes |
| nc20 | -5.9407813 | -3.8813461 | -5.2316081 | -5.0179118 | 1.046216 | -4.3174983 | -2.41 | 2 | 0.97516 | yes |
| nc39 | -5.0066897 | -4.9884984 | -3.7336388 | -4.5762756 | 0.7298016 | -10.054026 | -0.34 | 2 | 0.99513 | yes |
| nc3 | -5.3378143 | -4.3309654 | -2.4955 | -4.0547599 | 1.441147 | -3.118527 | -1.46 | 2 | 0.95536 | yes |
| nc4 | -2.9127083 | -0.0098432 | 0.568 | -0.7848505 | 1.8652908 | -0.4873598 | -0.26 | 2 | 0.66291 | no |
| nc5 | 2.2324073 | 7.0942707 | 3.1285261 | 4.1517347 | 2.5874014 | -1.1970954 | 5.94 | 2 | 0.82304 | no |
| nc6 | -2.8142792 | -11.503333 | -3.4210679 | -5.9128935 | 7.1786326 | -0.3770928 | -4.35 | 2 | 0.62882 | no |
| nc8 | -4.2342135 | -0.02447 | -1.6545328 | -1.9710721 | 2.1226476 | 1.2720634 | -3.53 | 2 | 0.16562 | no |
| nc9 | -3.3351765 | -0.663346 | -2.541357 | -2.1799598 | 1.372088 | 4.5697459 | -5.8 | 2 | 0.02235 | yes |
| nc14 | 0.8655942 | 7.3749781 | 1.7926528 | 3.3444084 | 3.5212183 | 2.299293 | -1.33 | 2 | 0.07411 | no |
| nc19 | -4.431303 | -7.9174738 | -5.755195 | -6.0346573 | 1.7598071 | -4.2269204 | -1.74 | 2 | 0.97417 | yes |
| nc28 | -5.9044927 | -8.4537621 | -7.5597426 | -7.3059991 | 1.2934384 | 0.9695137 | -8.03 | 2 | 0.21728 | no |
| nc29 | -1.5766493 | 5.110356 | 0.805 | 1.4462356 | 3.3893063 | -0.1501236 | 1.74 | 2 | 0.55278 | no |
| nc30 | -7.3428214 | -2.5178127 | -4.9763098 | -4.945648 | 2.4126505 | 1.2522768 | -6.69 | 2 | 0.16853 | no |
| nc36 | -2.5954641 | 4.2745342 | -2.5454741 | -0.2888013 | 3.9520435 | 1.2495867 | -3.14 | 2 | 0.16893 | no |
| nc47 | 0.6764933 | 5.1784007 | -0.554 | 1.7669647 | 3.0177724 | 2.5236384 | -2.63 | 2 | 0.063819 | no |
| nc52 | -3.1622715 | -1.1061325 | -3.4495 | -2.5726347 | 1.2781223 | -2.1311601 | -1 | 2 | 0.91662 | no |
| nc54 | -4.9575024 | 5.8240515 | -3.4855 | -0.8729836 | 5.8463157 | 0.7279246 | -3.33 | 2 | 0.27117 | no |
| nc56 | -5.1232402 | -4.006321 | -3.4535 | -4.1943537 | 0.8506029 | 1.2536175 | -4.81 | 2 | 0.16833 | no |
| nc61 | -5.5833333 | -10.911763 | -3.5315 | -6.6755322 | 3.8094284 | -0.620873 | -5.31 | 2 | 0.70099 | no |
| nc68 | -3.6867421 | -0.6753483 | -1.9583333 | -2.1068079 | 1.5111773 | -0.122419 | -2 | 2 | 0.54312 | no |
| nc76 | 1.7054838 | 7.9070498 | 1.0613333 | 3.5579556 | 3.7801716 | 2.4595634 | -1.81 | 2 | 0.066544 | no |
| nc78 | -6.6339604 | -8.1012561 | -5.7413333 | -6.8255166 | 1.1915658 | 1.0676393 | -7.56 | 2 | 0.19874 | no |

**Table S4. Detailed information on motif CPGDMM1**

| **ID** | **Strand** | **Motif Sequence** | **Motif start** | **Motif end** | **Putative base modification position** | **IPD ratio** | ***p* value** | ***p*< 0.01** |
| --- | --- | --- | --- | --- | --- | --- | --- | --- |
| 1 | N | TATAAATATCA | 106 | 116 | 117 | 1.61 | 0.007 | yes |
| 3 | N | TATATTGATAA | 3792 | 3802 | 3803 | 1.61 | 0.003 | yes |
| 5 | N | TATAAAGATCA | 6459 | 6469 | 6470 | 1.75 | 0.040 | no |
| 6 | N | TATATGTATCA | 7139 | 7149 | 7150 | 1.75 | 0.005 | yes |
| 8 | N | TATAGAAATAA | 8394 | 8404 | 8405 | 1.22 | 0.103 | no |
| 9 | N | TATAATAATAA | 8632 | 8642 | 8643 | 1.54 | 0.005 | yes |
| 12 | N | TATAGAAATTA | 16796 | 16806 | 16807 | 1.53 | 0.004 | yes |
| 18 | N | TATATATATAA | 30627 | 30637 | 30638 | 1.56 | 0.007 | yes |
| 19 | N | TATATATATTA | 30636 | 30646 | 30647 | 0.76 | 0.942 | no |
| 21 | N | TATAAGCATGA | 30947 | 30957 | 30958 | 0.90 | 0.777 | no |
| 23 | N | TATATGGATAA | 35049 | 35059 | 35060 | 1.40 | 0.012 | no |
| 25 | N | TATACCCATCA | 37694 | 37704 | 37705 | 1.36 | 0.023 | no |
| 27 | N | TATATTCATAA | 39729 | 39739 | 39740 | 1.50 | 0.008 | yes |
| 28 | N | TATATCAATAA | 41476 | 41486 | 41487 | 1.24 | 0.096 | no |
| 29 | N | TATATTCATAA | 42643 | 42653 | 42654 | 1.40 | 0.025 | no |
| 31 | N | TATATCAATGA | 44794 | 44804 | 44805 | 1.09 | 0.292 | no |
| 33 | N | TATATATATAA | 45805 | 45815 | 45816 | 1.51 | 0.006 | yes |
| 36 | N | TATATACATGA | 51471 | 51481 | 51482 | 1.64 | 0.004 | yes |
| 37 | N | TATAAATATCA | 51576 | 51586 | 51587 | 1.84 | 0.001 | yes |
| 40 | N | TATATAAATAA | 56258 | 56268 | 56269 | 1.90 | 2.49e-05 | yes |
| 41 | N | TATAAATATGA | 56514 | 56524 | 56525 | 1.11 | 0.273 | no |
| 44 | N | TATACTAATTA | 58581 | 58591 | 58592 | 1.18 | 0.189 | no |
| 45 | N | TATAAATATAA | 62738 | 62748 | 62749 | 1.82 | 5.80e-05 | yes |
| 48 | N | TATATATATAA | 62915 | 62925 | 62926 | 1.57 | 0.005 | yes |
| 52 | N | TATATTTATGA | 70444 | 70454 | 70455 | 1.66 | 0.001 | yes |
| 53 | N | TATATCAATTA | 70532 | 70542 | 70543 | 0.96 | 0.604 | no |
| 54 | N | TATAATCATAA | 72150 | 72160 | 72161 | 1.65 | 0.001 | yes |
| 55 | N | TATAATCATAA | 73220 | 73230 | 73231 | 1.12 | 0.255 | no |
| 58 | N | TATATTTATAA | 75017 | 75027 | 75028 | 1.52 | 0.009 | yes |
| 59 | N | TATATGAATGA | 75790 | 75800 | 75801 | 1.42 | 0.028 | no |
| 60 | N | TATATACATTA | 76724 | 76734 | 76735 | 1.16 | 0.163 | no |
| 61 | N | TATATTCATAA | 79242 | 79252 | 79253 | 2.07 | 5.44e-05 | yes |
| 63 | N | TATAGTGATAA | 82829 | 82839 | 82840 | 1.04 | 0.393 | no |
| 64 | N | TATAGATATCA | 83323 | 83333 | 83334 | 1.24 | 0.221 | no |
| 68 | N | TATATGCATCA | 87331 | 87341 | 87342 | 1.85 | 0.081 | no |
| 69 | N | TATATACATAA | 92593 | 92603 | 92604 | 1.82 | 0.118 | no |
| 71 | N | TATAATAATGA | 109764 | 109774 | 109775 | 1.30 | 0.060 | no |
| 73 | N | TATAGTGATAA | 110085 | 110095 | 110096 | 1.68 | 0.003 | yes |
| 74 | N | TATAGTCATGA | 110266 | 110276 | 110277 | 1.92 | 0.001 | yes |
| 76 | N | TATAGAAATTA | 111339 | 111349 | 111350 | 1.10 | 0.315 | no |
| 79 | N | TATAAAGATAA | 116502 | 116512 | 116513 | 1.02 | 0.462 | no |
| 82 | N | TATAATTATAA | 118002 | 118012 | 118013 | 2.37 | 8.25e-06 | yes |
| 84 | N | TATAGATATCA | 119377 | 119387 | 119388 | 1.30 | 0.042 | no |
| 85 | N | TATAAATATTA | 122253 | 122263 | 122264 | 1.60 | 0.019 | no |
| 87 | N | TATACTTATAA | 122717 | 122727 | 122728 | 1.22 | 0.200 | no |
| 88 | N | TATAGATATAA | 122822 | 122832 | 122833 | 1.07 | 0.354 | no |
| 89 | N | TATATTTATTA | 122863 | 122873 | 122874 | 1.20 | 0.155 | no |
| 90 | N | TATATAGATAA | 127942 | 127952 | 127953 | 0.32 | 0.999 | no |
| 93 | N | TATATGGATGA | 148447 | 148457 | 148458 | 0.67 | 0.889 | no |
| 94 | N | TATACCTATGA | 148781 | 148791 | 148792 | 1.25 | 0.300 | no |
| 95 | N | TATATATATGA | 150676 | 150686 | 150687 | 0.90 | 0.576 | no |
| 2 | P | TATAGAAATAA | 2787 | 2797 | 2786 | 1.47 | 0.019 | no |
| 4 | P | TATATCTATAA | 6197 | 6207 | 6196 | 1.69 | 0.036 | no |
| 7 | P | TATATAAATAA | 8189 | 8199 | 8188 | 2.10 | 3.26e-05 | yes |
| 10 | P | TATAGGGATCA | 11086 | 11096 | 11085 | 1.54 | 0.007 | yes |
| 11 | P | TATATCGATAA | 12476 | 12486 | 12475 | 1.22 | 0.130 | no |
| 13 | P | TATATATATCA | 19706 | 19716 | 19705 | 1.23 | 0.091 | no |
| 14 | P | TATATTTATTA | 20961 | 20971 | 20960 | 1.26 | 0.067 | no |
| 15 | P | TATATAAATGA | 24921 | 24931 | 24920 | 1.54 | 0.014 | no |
| 16 | P | TATAGAGATTA | 28041 | 28051 | 28040 | 1.07 | 0.324 | no |
| 17 | P | TATATATATAA | 30628 | 30638 | 30627 | 1.55 | 0.005 | yes |
| 20 | P | TATATGAATAA | 30954 | 30964 | 30953 | 0.92 | 0.688 | no |
| 22 | P | TATAGCTATCA | 31368 | 31378 | 31367 | 1.41 | 0.033 | no |
| 24 | P | TATAGAAATGA | 36166 | 36176 | 36165 | 1.11 | 0.265 | no |
| 26 | P | TATACATATGA | 37944 | 37954 | 37943 | 1.18 | 0.141 | no |
| 30 | P | TATACCAATGA | 44021 | 44031 | 44020 | 1.64 | 0.002 | yes |
| 32 | P | TATATATATAA | 45806 | 45816 | 45805 | 1.67 | 0.004 | yes |
| 34 | P | TATACGTATAA | 48511 | 48521 | 48510 | 1.46 | 0.011 | no |
| 35 | P | TATACTCATTA | 51194 | 51204 | 51193 | 0.55 | 0.998 | no |
| 38 | P | TATAAATATTA | 53666 | 53676 | 53665 | 0.87 | 0.794 | no |
| 39 | P | TATATATATGA | 54370 | 54380 | 54369 | 1.29 | 0.140 | no |
| 42 | P | TATATGAATAA | 57083 | 57093 | 57082 | 0.90 | 0.753 | no |
| 43 | P | TATAAATATAA | 58588 | 58598 | 58587 | 1.59 | 0.016 | no |
| 46 | P | TATAGGAATAA | 62819 | 62829 | 62818 | 1.17 | 0.155 | no |
| 47 | P | TATATATATAA | 62920 | 62930 | 62919 | 1.59 | 0.003 | yes |
| 49 | P | TATAGATATTA | 65057 | 65067 | 65056 | 0.95 | 0.634 | no |
| 50 | P | TATAATAATAA | 65785 | 65795 | 65784 | 1.59 | 0.003 | yes |
| 51 | P | TATATAAATCA | 67555 | 67565 | 67554 | 1.22 | 0.165 | no |
| 56 | P | TATAAGAATAA | 73551 | 73561 | 73550 | 1.03 | 0.419 | no |
| 57 | P | TATAAATATAA | 75018 | 75028 | 75017 | 2.07 | 6.64e-05 | yes |
| 62 | P | TATATATATCA | 81955 | 81965 | 81954 | 0.94 | 0.597 | no |
| 65 | P | TATATATATGA | 83345 | 83355 | 83344 | 1.05 | 0.362 | no |
| 66 | P | TATACCTATGA | 85240 | 85250 | 85239 | 1.32 | 0.019 | no |
| 67 | P | TATATGGATGA | 85574 | 85584 | 85573 | 1.45 | 0.002 | yes |
| 70 | P | TATATAGATAA | 106079 | 106089 | 106078 | 1.10 | 0.267 | no |
| 72 | P | TATAATTATCA | 110080 | 110090 | 110079 | 1.09 | 0.312 | no |
| 75 | P | TATACGAATGA | 110842 | 110852 | 110841 | 0.91 | 0.714 | no |
| 77 | P | TATAATTATAA | 113677 | 113687 | 113676 | 1.51 | 0.004 | yes |
| 78 | P | TATACATATTA | 116401 | 116411 | 116400 | 1.56 | 0.010 | yes |
| 80 | P | TATATTTATAA | 117997 | 118007 | 117996 | 2.24 | 1.24e-05 | yes |
| 81 | P | TATAATTATAA | 118003 | 118013 | 118002 | 1.75 | 0.004 | yes |
| 83 | P | TATATCAATTA | 119338 | 119348 | 119337 | 1.03 | 0.417 | no |
| 86 | P | TATAAGTATAA | 122718 | 122728 | 122717 | 1.34 | 0.077 | no |
| 91 | P | TATATACATAA | 141428 | 141438 | 141427 | 1.76 | 1.57e-06 | yes |
| 92 | P | TATATGCATCA | 146690 | 146700 | 146689 | 1.19 | 0.098 | no |
| 96 | P | TATAGATATCA | 150698 | 150708 | 150697 | 1.15 | 0.131 | no |
| 97 | P | TATAGTGATAA | 151192 | 151202 | 151191 | 1.55 | 0.002 | yes |

**Table S5. Detailed information on motif CPGDMM2**

| **ID** | **Strand** | **Motif Sequence** | **Motif start** | **Motif end** | **Putative base modification position** | **IPD ratio** | ***p* value** | ***p*< 0.01** |
| --- | --- | --- | --- | --- | --- | --- | --- | --- |
| 1 | N | TATAATGAT | 224 | 232 | 235 | 1.70 | 0.001 | yes |
| 2 | P | AGCAGTGAA | 590 | 599 | 587 | 1.04 | 0.436 | no |
| 3 | P | TGTAATGAA | 636 | 645 | 633 | 1.04 | 0.396 | no |
| 4 | P | AGCACTGAA | 812 | 821 | 809 | 1.25 | 0.058 | no |
| 5 | P | AGCAATGAA | 1289 | 1298 | 1286 | 0.84 | 0.843 | no |
| 6 | N | TGCAATGAA | 1792 | 1800 | 1803 | 1.40 | 0.059 | no |
| 7 | P | ATTATTGAT | 2455 | 2464 | 2452 | 1.49 | 0.029 | no |
| 8 | P | AATATTGAA | 3134 | 3143 | 3131 | 1.51 | 0.010 | yes |
| 9 | N | TCTAATGAT | 3307 | 3315 | 3318 | 1.30 | 0.051 | no |
| 10 | N | TATATTGAT | 3794 | 3802 | 3805 | 1.67 | 0.001 | yes |
| 11 | N | TTTAATGAA | 3859 | 3867 | 3870 | 0.95 | 0.632 | no |
| 12 | P | TCCATTGAT | 4102 | 4111 | 4099 | 1.65 | 6.72e-04 | yes |
| 13 | N | TGTATTGAA | 4660 | 4668 | 4671 | 1.37 | 0.059 | no |
| 14 | N | TCCAATGAA | 4946 | 4954 | 4957 | 1.25 | 0.143 | no |
| 15 | P | TTTAGTGAA | 5287 | 5296 | 5284 | 1.42 | 0.015 | no |
| 16 | N | TTTAATGAT | 6331 | 6339 | 6342 | 1.21 | 0.143 | no |
| 17 | N | ATTAGTGAT | 6504 | 6512 | 6515 | 0.76 | 0.933 | no |
| 18 | P | TTTATTGAT | 7172 | 7181 | 7169 | 1.11 | 0.317 | no |
| 19 | N | TGTAATGAT | 7179 | 7187 | 7190 | 1.13 | 0.254 | no |
| 20 | P | TACATTGAA | 7671 | 7680 | 7668 | 1.03 | 0.432 | no |
| 21 | P | TCTAATGAT | 8031 | 8040 | 8028 | 2.10 | 1.57e-05 | yes |
| 22 | P | AATAATGAT | 8598 | 8607 | 8595 | 0.87 | 0.813 | no |
| 23 | N | TATATTGAA | 8653 | 8661 | 8664 | 1.91 | 2.67e-04 | yes |
| 24 | P | ATTAGTGAA | 9763 | 9772 | 9760 | 0.93 | 0.661 | no |
| 25 | N | TTTACTGAA | 10119 | 10127 | 10130 | 0.85 | 0.853 | no |
| 26 | P | TCTAATGAA | 10222 | 10231 | 10219 | 1.37 | 0.009 | yes |
| 27 | P | AGCATTGAA | 10515 | 10524 | 10512 | 0.92 | 0.701 | no |
| 28 | N | ATTACTGAT | 10551 | 10559 | 10562 | 1.55 | 0.013 | no |
| 29 | N | TGTATTGAT | 12795 | 12803 | 12806 | 1.38 | 0.045 | no |
| 30 | N | TGTAGTGAT | 12807 | 12815 | 12818 | 1.10 | 0.304 | no |
| 31 | N | AATACTGAT | 12845 | 12853 | 12856 | 1.46 | 0.019 | no |
| 32 | N | TCCACTGAT | 13425 | 13433 | 13436 | 1.22 | 0.130 | no |
| 33 | P | ACCAATGAA | 13486 | 13495 | 13483 | 0.89 | 0.787 | no |
| 34 | P | ATTATTGAA | 13573 | 13582 | 13570 | 1.27 | 0.084 | no |
| 35 | N | AATAATGAT | 13912 | 13920 | 13923 | 1.05 | 0.397 | no |
| 36 | P | TTTATTGAT | 13990 | 13999 | 13987 | 1.93 | 5.10e-04 | yes |
| 37 | N | AATATTGAA | 14023 | 14031 | 14034 | 0.94 | 0.650 | no |
| 38 | P | TTCAATGAT | 14408 | 14417 | 14405 | 1.40 | 0.053 | no |
| 39 | N | ATCATTGAA | 14408 | 14416 | 14419 | 1.16 | 0.184 | no |
| 40 | P | ACCACTGAA | 14518 | 14527 | 14515 | 0.88 | 0.785 | no |
| 41 | N | ATCATTGAT | 15584 | 15592 | 15595 | 1.24 | 0.059 | no |
| 42 | P | ATCAATGAT | 15584 | 15593 | 15581 | 1.10 | 0.283 | no |
| 43 | N | TTTAATGAT | 16305 | 16313 | 16316 | 1.61 | 0.002 | yes |
| 44 | N | AATAATGAA | 17275 | 17283 | 17286 | 2.23 | 2.22e-04 | yes |
| 45 | P | AATATTGAA | 17393 | 17402 | 17390 | 1.51 | 0.013 | no |
| 46 | N | AGTATTGAA | 17962 | 17970 | 17973 | 1.17 | 0.178 | no |
| 47 | N | ACTATTGAA | 19305 | 19313 | 19316 | 1.67 | 9.60e-05 | yes |
| 48 | N | TTCAATGAA | 19383 | 19391 | 19394 | 1.13 | 0.183 | no |
| 49 | P | TTCATTGAA | 19383 | 19392 | 19380 | 1.01 | 0.465 | no |
| 50 | N | TCCATTGAA | 20169 | 20177 | 20180 | 1.22 | 0.120 | no |
| 51 | P | TGCATTGAA | 21223 | 21232 | 21220 | 0.92 | 0.705 | no |
| 52 | P | TGTAATGAA | 21509 | 21518 | 21506 | 1.39 | 0.020 | no |
| 53 | N | ATTAATGAA | 21805 | 21813 | 21816 | 1.60 | 0.002 | yes |
| 54 | P | AGTATTGAT | 22884 | 22893 | 22881 | 0.57 | 0.999 | no |
| 55 | P | TATAATGAA | 23322 | 23331 | 23319 | 1.61 | 7.04e-04 | yes |
| 56 | P | TCCAGTGAT | 23619 | 23628 | 23616 | 1.76 | 8.74e-04 | yes |
| 57 | P | ACTAATGAA | 24871 | 24880 | 24868 | 2.10 | 1.79e-06 | yes |
| 58 | N | TTTATTGAA | 25092 | 25100 | 25103 | 1.83 | 4.70e-05 | yes |
| 59 | P | AACATTGAT | 25401 | 25410 | 25398 | 1.14 | 0.188 | no |
| 60 | N | TCTAATGAA | 26374 | 26382 | 26385 | 1.04 | 0.423 | no |
| 61 | P | AGCACTGAT | 26771 | 26780 | 26768 | 0.88 | 0.773 | no |
| 62 | P | TTTACTGAA | 26795 | 26804 | 26792 | 1.11 | 0.284 | no |
| 63 | N | AGTATTGAT | 27498 | 27506 | 27509 | 1.10 | 0.279 | no |
| 64 | P | AATACTGAT | 27501 | 27510 | 27498 | 0.99 | 0.515 | no |
| 65 | P | TATATTGAT | 28834 | 28843 | 28831 | 1.47 | 0.033 | no |
| 66 | N | TCCATTGAA | 29325 | 29333 | 29336 | 1.82 | 0.002 | yes |
| 67 | N | TCTAATGAA | 30324 | 30332 | 30335 | 1.45 | 0.028 | no |
| 68 | N | TTCATTGAA | 30435 | 30443 | 30446 | 1.63 | 0.023 | no |
| 69 | P | TTCAATGAA | 30435 | 30444 | 30432 | 1.34 | 0.107 | no |
| 70 | N | TTTAATGAA | 31493 | 31501 | 31504 | 1.28 | 0.065 | no |
| 71 | N | TTTATTGAA | 31502 | 31510 | 31513 | 1.86 | 3.78e-04 | yes |
| 72 | N | TCTATTGAA | 31545 | 31553 | 31556 | 1.66 | 0.004 | yes |
| 73 | N | AGCAATGAA | 34023 | 34031 | 34034 | 0.84 | 0.853 | no |
| 74 | N | ATTATTGAA | 34047 | 34055 | 34058 | 1.14 | 0.189 | no |
| 75 | P | TTCAATGAA | 34824 | 34833 | 34821 | 1.48 | 0.038 | no |
| 76 | N | TTCATTGAA | 34824 | 34832 | 34835 | 1.39 | 0.049 | no |
| 77 | P | TCTACTGAT | 35038 | 35047 | 35035 | 0.98 | 0.554 | no |
| 78 | P | TACACTGAT | 35207 | 35216 | 35204 | 1.44 | 0.007 | yes |
| 79 | N | TCTAATGAA | 35602 | 35610 | 35613 | 1.24 | 0.107 | no |
| 80 | P | TCCATTGAT | 36922 | 36931 | 36919 | 1.80 | 8.18e-04 | yes |
| 81 | N | TTTAATGAA | 36991 | 36999 | 37002 | 1.21 | 0.142 | no |
| 82 | N | TACATTGAT | 37226 | 37234 | 37237 | 0.97 | 0.584 | no |
| 83 | P | AATAATGAA | 37298 | 37307 | 37295 | 0.96 | 0.573 | no |
| 84 | P | TCTAATGAA | 37636 | 37645 | 37633 | 1.62 | 0.003 | yes |
| 85 | N | ACTAATGAA | 38425 | 38433 | 38436 | 1.61 | 3.23e-04 | yes |
| 86 | P | ACCACTGAT | 38454 | 38463 | 38451 | 1.10 | 0.242 | no |
| 87 | P | TCCACTGAA | 39038 | 39047 | 39035 | 0.77 | 0.918 | no |
| 88 | N | ACTAGTGAA | 40643 | 40651 | 40654 | 1.96 | 5.04e-05 | yes |
| 89 | N | AATATTGAA | 40728 | 40736 | 40739 | 1.08 | 0.303 | no |
| 90 | N | ACCAGTGAT | 40916 | 40924 | 40927 | 1.79 | 1.92e-04 | yes |
| 91 | N | ATCAATGAT | 41444 | 41452 | 41455 | 1.70 | 0.003 | yes |
| 92 | P | ATCATTGAT | 41444 | 41453 | 41441 | 0.70 | 0.983 | no |
| 93 | P | TATATTGAA | 41467 | 41476 | 41464 | 1.11 | 0.274 | no |
| 94 | P | ATTATTGAT | 41481 | 41490 | 41478 | 2.12 | 1.45e-04 | yes |
| 95 | P | TATATTGAA | 41491 | 41500 | 41488 | 2.38 | 1.18e-06 | yes |
| 96 | P | AATATTGAA | 41498 | 41507 | 41495 | 1.20 | 0.163 | no |
| 97 | P | TATAATGAA | 41693 | 41702 | 41690 | 1.59 | 0.010 | yes |
| 98 | N | TATATTGAA | 41931 | 41939 | 41942 | 1.03 | 0.435 | no |
| 99 | N | TGTAATGAA | 42200 | 42208 | 42211 | 1.89 | 9.74e-04 | yes |
| 100 | N | TCTAATGAT | 42293 | 42301 | 42304 | 1.20 | 0.150 | no |
| 101 | P | TATAGTGAA | 42751 | 42760 | 42748 | 1.70 | 2.96e-04 | yes |
| 102 | N | TTTATTGAA | 43671 | 43679 | 43682 | 1.80 | 0.002 | yes |
| 103 | N | TTTATTGAT | 43788 | 43796 | 43799 | 1.37 | 0.032 | no |
| 104 | P | ACCAATGAA | 44030 | 44039 | 44027 | 1.81 | 2.30e-04 | yes |
| 105 | P | TTCAGTGAT | 44374 | 44383 | 44371 | 1.48 | 0.026 | no |
| 106 | N | ATCACTGAA | 44374 | 44382 | 44385 | 1.35 | 0.190 | no |
| 107 | P | TTCATTGAT | 44799 | 44808 | 44796 | 1.13 | 0.245 | no |
| 108 | N | ATCAATGAA | 44799 | 44807 | 44810 | 1.14 | 0.247 | no |
| 109 | N | TTTAGTGAA | 45253 | 45261 | 45264 | 1.14 | 0.201 | no |
| 110 | N | AATATTGAA | 45912 | 45920 | 45923 | 1.03 | 0.421 | no |
| 111 | P | TATATTGAA | 46546 | 46555 | 46543 | 2.08 | 2.18e-05 | yes |
| 112 | P | TCTATTGAA | 46991 | 47000 | 46988 | 1.66 | 0.005 | yes |
| 113 | P | AACAATGAA | 47200 | 47209 | 47197 | 1.17 | 0.290 | no |
| 114 | P | ACCATTGAT | 47457 | 47466 | 47454 | 1.19 | 0.097 | no |
| 115 | N | AACAATGAA | 49191 | 49199 | 49202 | 1.18 | 0.138 | no |
| 116 | P | ATTAGTGAA | 49287 | 49296 | 49284 | 1.49 | 0.006 | yes |
| 117 | N | TTTATTGAA | 49301 | 49309 | 49312 | 2.01 | 4.43e-05 | yes |
| 118 | N | TTCATTGAA | 50699 | 50707 | 50710 | 1.41 | 0.047 | no |
| 119 | P | TTCAATGAA | 50699 | 50708 | 50696 | 1.00 | 0.501 | no |
| 120 | N | AATAATGAA | 51010 | 51018 | 51021 | 0.81 | 0.888 | no |
| 121 | P | AACAGTGAT | 52084 | 52093 | 52081 | 0.98 | 0.561 | no |
| 122 | N | AGTACTGAA | 52890 | 52898 | 52901 | 1.00 | 0.501 | no |
| 123 | N | ATTAATGAA | 53133 | 53141 | 53144 | 1.31 | 0.037 | no |
| 124 | P | ACCAATGAT | 53721 | 53730 | 53718 | 1.07 | 0.330 | no |
| 125 | P | TATAATGAT | 54054 | 54063 | 54051 | 1.36 | 0.060 | no |
| 126 | N | AATAATGAA | 54131 | 54139 | 54142 | 0.98 | 0.555 | no |
| 127 | P | AATAATGAT | 54397 | 54406 | 54394 | 1.38 | 0.032 | no |
| 128 | N | TATATTGAT | 54873 | 54881 | 54884 | 2.20 | 5.74e-07 | yes |
| 129 | P | TATAGTGAA | 56527 | 56536 | 56524 | 1.31 | 0.050 | no |
| 130 | P | AGTAGTGAT | 56578 | 56587 | 56575 | 0.81 | 0.915 | no |
| 131 | P | ACTAGTGAA | 56813 | 56822 | 56810 | 1.78 | 1.96e-04 | yes |
| 132 | P | TACAGTGAT | 56891 | 56900 | 56888 | 1.57 | 7.19e-04 | yes |
| 133 | P | TGTATTGAT | 57215 | 57224 | 57212 | 0.98 | 0.558 | no |
| 134 | P | AATAGTGAA | 57290 | 57299 | 57287 | 1.18 | 0.134 | no |
| 135 | P | AACAGTGAT | 57332 | 57341 | 57329 | 1.11 | 0.235 | no |
| 136 | P | TCTAGTGAT | 57359 | 57368 | 57356 | 1.32 | 0.030 | no |
| 137 | P | AACATTGAA | 58039 | 58048 | 58036 | 1.16 | 0.219 | no |
| 138 | P | TCTAATGAA | 58515 | 58524 | 58512 | 1.16 | 0.229 | no |
| 139 | N | TTTATTGAA | 59204 | 59212 | 59215 | 1.57 | 0.005 | yes |
| 140 | P | AATAATGAT | 59985 | 59994 | 59982 | 2.06 | 9.33e-06 | yes |
| 141 | P | ACTAATGAT | 60844 | 60853 | 60841 | 1.00 | 0.505 | no |
| 142 | P | TTCAATGAA | 61006 | 61015 | 61003 | 1.96 | 0.003 | yes |
| 143 | N | TTCATTGAA | 61006 | 61014 | 61017 | 0.86 | 0.771 | no |
| 144 | P | ACCAATGAT | 61212 | 61221 | 61209 | 1.29 | 0.108 | no |
| 145 | P | TATAGTGAA | 61698 | 61707 | 61695 | 1.49 | 0.010 | yes |
| 146 | P | ACCAATGAA | 63038 | 63047 | 63035 | 1.51 | 0.026 | no |
| 147 | N | TTTACTGAT | 64085 | 64093 | 64096 | 1.11 | 0.244 | no |
| 148 | N | TTCAGTGAT | 64099 | 64107 | 64110 | 1.37 | 0.022 | no |
| 149 | P | ATCACTGAA | 64099 | 64108 | 64096 | 0.92 | 0.692 | no |
| 150 | N | TATACTGAT | 64165 | 64173 | 64176 | 1.25 | 0.070 | no |
| 151 | P | TACAATGAA | 64304 | 64313 | 64301 | 2.13 | 9.69e-06 | yes |
| 152 | P | TGTAATGAT | 64687 | 64696 | 64684 | 1.04 | 0.412 | no |
| 153 | P | TCTATTGAT | 66017 | 66026 | 66014 | 1.89 | 3.34e-04 | yes |
| 154 | N | TATAATGAA | 66071 | 66079 | 66082 | 1.58 | 0.007 | yes |
| 155 | P | TGCATTGAA | 66811 | 66820 | 66808 | 1.52 | 0.026 | no |
| 156 | P | ATTAGTGAA | 67014 | 67023 | 67011 | 1.25 | 0.166 | no |
| 157 | P | TTTATTGAA | 67330 | 67339 | 67327 | 1.05 | 0.416 | no |
| 158 | N | TTTATTGAT | 68077 | 68085 | 68088 | 1.18 | 0.180 | no |
| 159 | N | AACATTGAT | 68176 | 68184 | 68187 | 1.59 | 5.97e-04 | yes |
| 160 | N | ACCAGTGAA | 68551 | 68559 | 68562 | 1.52 | 0.002 | yes |
| 161 | N | ATCAGTGAT | 68839 | 68847 | 68850 | 1.06 | 0.389 | no |
| 162 | P | ATCACTGAT | 68839 | 68848 | 68836 | 0.92 | 0.694 | no |
| 163 | P | TATAATGAA | 69213 | 69222 | 69210 | 2.38 | 5.36e-08 | yes |
| 164 | N | TACATTGAT | 70095 | 70103 | 70106 | 1.67 | 8.39e-04 | yes |
| 165 | P | TCTAATGAT | 70958 | 70967 | 70955 | 1.37 | 0.045 | no |
| 166 | P | TCTACTGAA | 71124 | 71133 | 71121 | 0.82 | 0.900 | no |
| 167 | P | TGTATTGAA | 71257 | 71266 | 71254 | 1.51 | 0.020 | no |
| 168 | P | AATATTGAA | 71921 | 71930 | 71918 | 1.12 | 0.260 | no |
| 169 | P | TATAGTGAT | 72455 | 72464 | 72452 | 1.27 | 0.044 | no |
| 170 | P | ACCATTGAA | 73404 | 73413 | 73401 | 0.89 | 0.786 | no |
| 171 | P | TCCATTGAT | 73452 | 73461 | 73449 | 1.13 | 0.236 | no |
| 172 | N | TTCATTGAA | 73542 | 73550 | 73553 | 1.90 | 6.41e-04 | yes |
| 173 | P | TTCAATGAA | 73542 | 73551 | 73539 | 1.10 | 0.269 | no |
| 174 | P | TCTATTGAT | 73707 | 73716 | 73704 | 1.29 | 0.043 | no |
| 175 | N | ATTAGTGAT | 74073 | 74081 | 74084 | 2.48 | 2.16e-09 | yes |
| 176 | N | TGTATTGAA | 74587 | 74595 | 74598 | 1.28 | 0.075 | no |
| 177 | P | TCCAATGAT | 74990 | 74999 | 74987 | 1.00 | 0.497 | no |
| 178 | P | ATTATTGAA | 75117 | 75126 | 75114 | 1.02 | 0.458 | no |
| 179 | P | ATTATTGAT | 75242 | 75251 | 75239 | 1.01 | 0.469 | no |
| 180 | P | TATAATGAA | 75420 | 75429 | 75417 | 1.30 | 0.043 | no |
| 181 | N | AGTATTGAA | 75437 | 75445 | 75448 | 1.24 | 0.112 | no |
| 182 | P | TGCATTGAT | 75486 | 75495 | 75483 | 0.98 | 0.549 | no |
| 183 | P | TACATTGAA | 75788 | 75797 | 75785 | 1.24 | 0.127 | no |
| 184 | P | ATCAATGAT | 76119 | 76128 | 76116 | 0.89 | 0.766 | no |
| 185 | N | ATCATTGAT | 76119 | 76127 | 76130 | 0.87 | 0.810 | no |
| 186 | N | TTTATTGAT | 76791 | 76799 | 76802 | 1.12 | 0.223 | no |
| 187 | N | AGCATTGAA | 76807 | 76815 | 76818 | 0.79 | 0.940 | no |
| 188 | N | TCTAATGAA | 77341 | 77349 | 77352 | 1.31 | 0.049 | no |
| 189 | N | AACAGTGAA | 77667 | 77675 | 77678 | 1.55 | 0.003 | yes |
| 190 | P | ACCATTGAA | 77909 | 77918 | 77906 | 1.03 | 0.429 | no |
| 191 | N | ATTACTGAA | 78608 | 78616 | 78619 | 1.65 | 7.50e-04 | yes |
| 192 | P | AGTAGTGAA | 80605 | 80614 | 80602 | 1.34 | 0.028 | no |
| 193 | P | AGTATTGAT | 80836 | 80845 | 80833 | 1.00 | 0.486 | no |
| 194 | N | ATCAATGAT | 80903 | 80911 | 80914 | 1.31 | 0.063 | no |
| 195 | P | ATCATTGAT | 80903 | 80912 | 80900 | 1.20 | 0.212 | no |
| 196 | N | AGCAATGAA | 81512 | 81520 | 81523 | 0.95 | 0.617 | no |
| 197 | P | ACTAATGAA | 82414 | 82423 | 82411 | 1.31 | 0.072 | no |
| 198 | N | TACAATGAT | 82615 | 82623 | 82626 | 1.52 | 0.016 | no |
| 199 | P | TTTAGTGAA | 82732 | 82741 | 82729 | 0.92 | 0.713 | no |
| 200 | N | TATAGTGAT | 82838 | 82846 | 82849 | 1.36 | 0.064 | no |
| 201 | P | ACTACTGAA | 83743 | 83752 | 83740 | 0.95 | 0.645 | no |
| 202 | P | ATCAGTGAT | 83821 | 83830 | 83818 | 0.97 | 0.570 | no |
| 203 | N | ATCACTGAT | 83821 | 83829 | 83832 | 0.51 | 0.971 | no |
| 204 | N | ACTATTGAT | 83880 | 83888 | 83891 | 0.46 | 0.998 | no |
| 205 | P | TATATTGAT | 84524 | 84533 | 84521 | 1.64 | 1.94e-05 | yes |
| 206 | P | TCCATTGAT | 84704 | 84713 | 84701 | 1.39 | 0.002 | yes |
| 207 | N | TGCATTGAT | 84894 | 84902 | 84905 | 1.34 | 0.145 | no |
| 208 | N | TTCATTGAT | 84914 | 84922 | 84925 | 1.52 | 0.046 | no |
| 209 | P | ATCAATGAA | 84914 | 84923 | 84911 | 1.10 | 0.256 | no |
| 210 | P | TCTAATGAA | 85503 | 85512 | 85500 | 1.52 | 3.49e-04 | yes |
| 211 | P | TCTATTGAT | 86832 | 86841 | 86829 | 1.69 | 1.95e-05 | yes |
| 212 | N | TATATTGAT | 87058 | 87066 | 87069 | 1.35 | 0.175 | no |
| 213 | P | TGTATTGAA | 87122 | 87131 | 87119 | 1.11 | 0.202 | no |
| 214 | P | TTTAATGAA | 87140 | 87149 | 87137 | 1.42 | 0.004 | yes |
| 215 | P | TATAATGAA | 87221 | 87230 | 87218 | 1.72 | 2.65e-06 | yes |
| 216 | N | AATATTGAT | 87445 | 87453 | 87456 | 0.89 | 0.577 | no |
| 217 | N | TCCATTGAT | 87762 | 87770 | 87773 | 0.89 | 0.603 | no |
| 218 | P | TTCATTGAT | 87932 | 87941 | 87929 | 1.30 | 0.020 | no |
| 219 | N | ATCAATGAA | 87932 | 87940 | 87943 | 1.10 | 0.432 | no |
| 220 | N | ATTATTGAT | 88044 | 88052 | NA | NA | NA | NA |
| 221 | P | ACCATTGAT | 88859 | 88868 | 88856 | 1.66 | 2.40e-05 | yes |
| 222 | P | TTCATTGAT | 89330 | 89339 | 89327 | 1.68 | 2.03e-04 | yes |
| 223 | N | ATCAATGAA | 89330 | 89338 | 89341 | 1.04 | 0.446 | no |
| 224 | P | AACAGTGAT | 89400 | 89409 | 89397 | 0.91 | 0.690 | no |
| 225 | P | TCTATTGAA | 89597 | 89606 | 89594 | 1.70 | 9.58e-05 | yes |
| 226 | N | TGTATTGAA | 89613 | 89621 | 89624 | 0.40 | 0.978 | no |
| 227 | P | TTTAGTGAT | 89768 | 89777 | 89765 | 0.96 | 0.623 | no |
| 228 | P | AATATTGAT | 89928 | 89937 | 89925 | 1.58 | 2.34e-04 | yes |
| 229 | P | AATATTGAT | 89946 | 89955 | 89943 | 1.35 | 0.011 | no |
| 230 | N | AATACTGAT | 91654 | 91662 | 91665 | 0.75 | 0.816 | no |
| 231 | P | ATTATTGAA | 92101 | 92110 | 92098 | 1.36 | 0.011 | no |
| 232 | P | TCTATTGAT | 92530 | 92539 | 92527 | 1.00 | 0.509 | no |
| 233 | N | TCTATTGAA | 92649 | 92657 | 92660 | 1.17 | 0.393 | no |
| 234 | P | TTCATTGAT | 93046 | 93055 | 93043 | 1.49 | 0.002 | yes |
| 235 | N | ATCAATGAA | 93046 | 93054 | 93057 | 0.73 | 0.867 | no |
| 236 | P | AGCAATGAT | 93600 | 93609 | 93597 | 1.59 | 1.58e-04 | yes |
| 237 | N | ACCACTGAT | 93970 | 93978 | 93981 | 0.44 | 0.994 | no |
| 238 | P | TCCATTGAT | 94460 | 94469 | 94457 | 1.64 | 3.44e-05 | yes |
| 239 | P | TACAGTGAT | 94503 | 94512 | 94500 | 1.88 | 2.24e-07 | yes |
| 240 | P | TGTATTGAT | 94554 | 94563 | 94551 | 1.35 | 0.015 | no |
| 241 | N | AATAGTGAA | 94570 | 94578 | 94581 | 1.11 | 0.420 | no |
| 242 | N | TACATTGAA | 94857 | 94865 | 94868 | 1.00 | 0.500 | no |
| 243 | N | AGCAATGAT | 96272 | 96280 | 96283 | 0.74 | 0.771 | no |
| 244 | N | AATATTGAA | 96368 | 96376 | NA | NA | NA | NA |
| 245 | P | AATATTGAA | 96371 | 96380 | 96368 | 1.27 | 0.036 | no |
| 246 | P | AGCAGTGAT | 96715 | 96724 | 96712 | 1.03 | 0.424 | no |
| 247 | N | TCCAATGAA | 96890 | 96898 | 96901 | 2.22 | 0.128 | no |
| 248 | P | ACCATTGAA | 97273 | 97282 | 97270 | 1.21 | 0.102 | no |
| 249 | N | TACACTGAT | 100804 | 100812 | 100815 | 1.49 | 0.282 | no |
| 250 | N | TGCACTGAT | 101163 | 101171 | 101174 | 0.35 | 0.985 | no |
| 251 | P | TCTATTGAT | 101410 | 101419 | 101407 | 1.44 | 0.002 | yes |
| 252 | P | AGTAATGAT | 101643 | 101652 | 101640 | 0.71 | 0.992 | no |
| 253 | P | TGCAGTGAT | 105557 | 105566 | 105554 | 1.25 | 0.041 | no |
| 254 | N | AACAATGAA | 105656 | 105664 | 105667 | 1.15 | 0.258 | no |
| 255 | N | TGTAATGAT | 106404 | 106412 | 106415 | 1.00 | 0.500 | no |
| 256 | N | TATAATGAA | 107009 | 107017 | 107020 | 2.34 | 0.016 | no |
| 257 | N | AACATTGAA | 107565 | 107573 | 107576 | 1.52 | 0.047 | no |
| 258 | N | AACATTGAA | 107626 | 107634 | 107637 | 1.39 | 0.108 | no |
| 259 | P | TCTAATGAA | 108175 | 108184 | 108172 | 1.55 | 0.005 | yes |
| 260 | N | AATAATGAA | 108936 | 108944 | 108947 | 1.90 | 8.02e-06 | yes |
| 261 | P | TCCATTGAA | 109327 | 109336 | 109324 | 0.99 | 0.531 | no |
| 262 | N | TATAATGAA | 109555 | 109563 | 109566 | 2.36 | 2.91e-07 | yes |
| 263 | N | AATAATGAA | 109770 | 109778 | 109781 | 1.39 | 0.037 | no |
| 264 | P | AGTACTGAA | 110031 | 110040 | 110028 | 1.21 | 0.122 | no |
| 265 | N | TATAGTGAT | 110094 | 110102 | 110105 | 1.20 | 0.152 | no |
| 266 | P | AGTAGTGAT | 110124 | 110133 | 110121 | 1.31 | 0.059 | no |
| 267 | P | AACAATGAA | 111630 | 111639 | 111627 | 1.26 | 0.214 | no |
| 268 | P | TATAATGAA | 111852 | 111861 | 111849 | 1.84 | 0.008 | yes |
| 269 | P | AATAATGAA | 111862 | 111871 | 111859 | 1.78 | 0.005 | yes |
| 270 | N | AATAATGAT | 112372 | 112380 | 112383 | 1.55 | 0.007 | yes |
| 271 | N | TACAATGAA | 113068 | 113076 | 113079 | 1.03 | 0.444 | no |
| 272 | N | TTTATTGAT | 113122 | 113130 | 113133 | 1.83 | 0.003 | yes |
| 273 | P | ACTACTGAA | 113498 | 113507 | 113495 | 0.97 | 0.586 | no |
| 274 | N | TCTACTGAA | 114062 | 114070 | 114073 | 1.20 | 0.122 | no |
| 275 | P | AGTAGTGAT | 114344 | 114353 | 114341 | 1.03 | 0.422 | no |
| 276 | P | TCCAATGAA | 114403 | 114412 | 114400 | 1.25 | 0.095 | no |
| 277 | P | TTTACTGAA | 115002 | 115011 | 114999 | 0.74 | 0.979 | no |
| 278 | P | TACAATGAA | 115258 | 115267 | 115255 | 1.18 | 0.200 | no |
| 279 | N | ATTAATGAT | 115570 | 115578 | 115581 | 0.77 | 0.970 | no |
| 280 | P | ATTATTGAA | 115836 | 115845 | 115833 | 1.06 | 0.369 | no |
| 281 | P | ATTAGTGAA | 115986 | 115995 | 115983 | 1.42 | 0.033 | no |
| 282 | P | ATCATTGAA | 116013 | 116022 | 116010 | 1.13 | 0.204 | no |
| 283 | N | TTCAATGAT | 116013 | 116021 | 116024 | 1.08 | 0.334 | no |
| 284 | N | AGTATTGAT | 116903 | 116911 | 116914 | 1.25 | 0.077 | no |
| 285 | P | TACAGTGAA | 117284 | 117293 | 117281 | 1.33 | 0.043 | no |
| 286 | P | AATAATGAA | 117566 | 117575 | 117563 | 1.03 | 0.428 | no |
| 287 | N | AGCAGTGAT | 117840 | 117848 | 117851 | 1.02 | 0.467 | no |
| 288 | P | ATTATTGAA | 119013 | 119022 | 119010 | 1.21 | 0.215 | no |
| 289 | P | AATACTGAA | 119060 | 119069 | 119057 | 1.20 | 0.151 | no |
| 290 | N | ATTAGTGAA | 119742 | 119750 | 119753 | 1.38 | 0.060 | no |
| 291 | P | TGCATTGAT | 120475 | 120484 | 120472 | 0.83 | 0.888 | no |
| 292 | P | ATTACTGAA | 120895 | 120904 | 120892 | 0.76 | 0.962 | no |
| 293 | N | ATTACTGAT | 120958 | 120966 | 120969 | 1.05 | 0.383 | no |
| 294 | N | ATTAATGAA | 121461 | 121469 | 121472 | 0.94 | 0.652 | no |
| 295 | N | AGTATTGAT | 121848 | 121856 | 121859 | 0.75 | 0.968 | no |
| 296 | P | ATTATTGAT | 121994 | 122003 | 121991 | 1.47 | 0.014 | no |
| 297 | N | ATTAATGAA | 122255 | 122263 | 122266 | 1.31 | 0.077 | no |
| 298 | N | TCTACTGAA | 122861 | 122869 | 122872 | 1.44 | 0.034 | no |
| 299 | N | AATAATGAA | 123380 | 123388 | 123391 | 1.80 | 1.60e-04 | yes |
| 300 | N | TTTATTGAT | 123401 | 123409 | 123412 | 1.09 | 0.289 | no |
| 301 | P | ATTAATGAT | 123455 | 123464 | 123452 | 1.02 | 0.441 | no |
| 302 | P | TTCAATGAA | 123491 | 123500 | 123488 | 1.23 | 0.088 | no |
| 303 | N | TTCATTGAA | 123491 | 123499 | 123502 | 0.94 | 0.617 | no |
| 304 | N | TCTAATGAA | 124131 | 124139 | 124142 | 1.30 | 0.140 | no |
| 305 | N | AATAGTGAT | 124823 | 124831 | 124834 | 1.26 | 0.085 | no |
| 306 | P | TTTACTGAT | 124970 | 124979 | 124967 | 1.37 | 0.052 | no |
| 307 | P | TTTACTGAT | 125144 | 125153 | 125141 | 1.10 | 0.287 | no |
| 308 | N | TGTAATGAA | 125543 | 125551 | 125554 | 1.25 | 0.090 | no |
| 309 | N | TCTAATGAA | 125862 | 125870 | 125873 | 1.14 | 0.231 | no |
| 310 | P | AACATTGAA | 126411 | 126420 | 126408 | 1.23 | 0.072 | no |
| 311 | P | AACATTGAA | 126472 | 126481 | 126469 | 1.03 | 0.418 | no |
| 312 | P | TATAATGAA | 127028 | 127037 | 127025 | 1.92 | 5.56e-07 | yes |
| 313 | P | TGTAATGAT | 127633 | 127642 | 127630 | 1.20 | 0.084 | no |
| 314 | P | AACAATGAA | 128381 | 128390 | 128378 | 1.11 | 0.232 | no |
| 315 | N | TGCAGTGAT | 128480 | 128488 | 128491 | 1.82 | 0.080 | no |
| 316 | N | AGTAATGAT | 132394 | 132402 | 132405 | 2.39 | 0.104 | no |
| 317 | N | TCTATTGAT | 132627 | 132635 | 132638 | 0.62 | 0.913 | no |
| 318 | P | TGCACTGAT | 132874 | 132883 | 132871 | 1.26 | 0.048 | no |
| 319 | P | TACACTGAT | 133233 | 133242 | 133230 | 1.36 | 0.007 | yes |
| 320 | N | ACCATTGAA | 136764 | 136772 | NA | NA | NA | NA |
| 321 | P | TCCAATGAA | 137147 | 137156 | 137144 | 1.25 | 0.052 | no |
| 322 | N | AGCAGTGAT | 137322 | 137330 | 137333 | 0.91 | 0.644 | no |
| 323 | N | AATATTGAA | 137666 | 137674 | 137677 | 2.13 | 0.187 | no |
| 324 | P | AATATTGAA | 137669 | 137678 | 137666 | 1.39 | 0.005 | yes |
| 325 | P | AGCAATGAT | 137765 | 137774 | 137762 | 1.05 | 0.338 | no |
| 326 | P | TACATTGAA | 139180 | 139189 | 139177 | 1.24 | 0.062 | no |
| 327 | P | AATAGTGAA | 139467 | 139476 | 139464 | 1.04 | 0.389 | no |
| 328 | N | TGTATTGAT | 139483 | 139491 | 139494 | 1.12 | 0.394 | no |
| 329 | N | TACAGTGAT | 139534 | 139542 | 139545 | 0.48 | 0.953 | no |
| 330 | N | TCCATTGAT | 139577 | 139585 | 139588 | 0.55 | 0.973 | no |
| 331 | P | ACCACTGAT | 140067 | 140076 | 140064 | 0.95 | 0.676 | no |
| 332 | N | AGCAATGAT | 140437 | 140445 | 140448 | 3.53 | 0.009 | yes |
| 333 | P | ATCAATGAA | 140991 | 141000 | 140988 | 1.26 | 0.035 | no |
| 334 | N | TTCATTGAT | 140991 | 140999 | NA | NA | NA | NA |
| 335 | P | TCTATTGAA | 141388 | 141397 | 141385 | 1.30 | 0.017 | no |
| 336 | N | TCTATTGAT | 141507 | 141515 | 141518 | 1.11 | 0.345 | no |
| 337 | N | ATTATTGAA | 141936 | 141944 | 141947 | 5.29 | 0.025 | no |
| 338 | P | AATACTGAT | 142383 | 142392 | 142380 | 1.35 | 0.006 | yes |
| 339 | N | AATATTGAT | 144091 | 144099 | 144102 | 1.29 | 0.309 | no |
| 340 | N | AATATTGAT | 144109 | 144117 | 144120 | 2.03 | 0.028 | no |
| 341 | N | TTTAGTGAT | 144269 | 144277 | 144280 | 0.87 | 0.638 | no |
| 342 | P | TGTATTGAA | 144424 | 144433 | 144421 | 1.52 | 0.002 | yes |
| 343 | N | TCTATTGAA | 144440 | 144448 | 144451 | 1.46 | 0.243 | no |
| 344 | N | AACAGTGAT | 144637 | 144645 | 144648 | 0.45 | 0.947 | no |
| 345 | P | ATCAATGAA | 144707 | 144716 | 144704 | 1.22 | 0.077 | no |
| 346 | N | TTCATTGAT | 144707 | 144715 | 144718 | 1.11 | 0.425 | no |
| 347 | N | ACCATTGAT | 145178 | 145186 | 145189 | 3.52 | 0.063 | no |
| 348 | P | ATTATTGAT | 145993 | 146002 | 145990 | 1.67 | 5.23e-05 | yes |
| 349 | P | ATCAATGAA | 146105 | 146114 | 146102 | 1.54 | 0.003 | yes |
| 350 | N | TTCATTGAT | 146105 | 146113 | NA | NA | NA | NA |
| 351 | P | TCCATTGAT | 146275 | 146284 | 146272 | 1.82 | 9.14e-04 | yes |
| 352 | P | AATATTGAT | 146592 | 146601 | 146589 | 1.05 | 0.382 | no |
| 353 | N | TATAATGAA | 146816 | 146824 | 146827 | 1.45 | 0.172 | no |
| 354 | N | TTTAATGAA | 146897 | 146905 | 146908 | 1.89 | 0.079 | no |
| 355 | N | TGTATTGAA | 146915 | 146923 | 146926 | 1.30 | 0.260 | no |
| 356 | P | TATATTGAT | 146979 | 146988 | 146976 | 1.58 | 8.85e-05 | yes |
| 357 | N | TCTATTGAT | 147205 | 147213 | 147216 | 1.29 | 0.271 | no |
| 358 | N | TCTAATGAA | 148534 | 148542 | 148545 | 1.93 | 0.110 | no |
| 359 | P | TTCATTGAT | 149123 | 149132 | 149120 | 1.89 | 1.23e-06 | yes |
| 360 | N | ATCAATGAA | 149123 | 149131 | 149134 | 1.32 | 0.284 | no |
| 361 | P | TGCATTGAT | 149143 | 149152 | 149140 | 1.11 | 0.195 | no |
| 362 | N | TCCATTGAT | 149333 | 149341 | 149344 | 1.50 | 0.092 | no |
| 363 | N | TATATTGAT | 149513 | 149521 | 149524 | 1.54 | 0.318 | no |
| 364 | P | ACTATTGAT | 150157 | 150166 | 150154 | 1.68 | 1.28e-04 | yes |
| 365 | P | ATCACTGAT | 150216 | 150225 | 150213 | 1.24 | 0.156 | no |
| 366 | N | ATCAGTGAT | 150216 | 150224 | 150227 | 0.49 | 0.980 | no |
| 367 | N | ACTACTGAA | 150294 | 150302 | 150305 | 1.44 | 0.073 | no |
| 368 | P | TATAGTGAT | 151199 | 151208 | 151196 | 2.09 | 2.12e-07 | yes |
| 369 | N | TTTAGTGAA | 151305 | 151313 | 151316 | 1.35 | 0.223 | no |

**Table S6. Associations between DNA modification site (DMS) and downstream ncRNA expression**

| **DMS ID** | **DMS Pos** | **Position of ncRNA Genes Adjacent to DMS** | **DMS**  **Strand** | **Motif Sequence** | **IPD Ratio** | ***p* value** | ***p*< 0.01** | **ncRNA Name** | **Expression Abundance** | **Distance between DMM1 and ncRNA Genes** | **Category Based on the Distance** |
| --- | --- | --- | --- | --- | --- | --- | --- | --- | --- | --- | --- |
| dmsite30 | 44020 | 44037 | P | TATACCAATGA | 1.64 | 0.002 | yes | nc39 | 4.17654 | 17 | <400 |
| dmsite81 | 118002 | 118049 | P | TATAATTATAA | 1.75 | 0.004 | yes | nc105 | 3.854612 | 47 | <400 |
| dmsite80 | 117996 | 118049 | P | TATATTTATAA | 2.24 | 1.24e-05 | yes | nc105 | 3.854612 | 53 | <400 |
| dmsite72 | 110079 | 110141 | P | TATAATTATCA | 1.09 | 0.312 | no | nc99 | 2.854612 | 62 | <400 |
| dmsite7 | 8188 | 8278 | P | TATATAAATAA | 2.10 | 3.26e-05 | yes | nc7 | 4.17654 | 90 | <400 |
| dmsite76 | 111350 | 111234 | N | TATAGAAATTA | 1.10 | 0.315 | no | nc100 | 1.854612 | 116 | <400 |
| dmsite45 | 62749 | 62606 | N | TATAAATATAA | 1.82 | 0.000 | yes | nc68 | 3.439575 | 143 | <400 |
| dmsite86 | 122717 | 122874 | P | TATAAGTATAA | 1.34 | 0.077 | no | nc109 | 1.854612 | 157 | <400 |
| dmsite77 | 113676 | 113867 | P | TATAATTATAA | 1.51 | 0.004 | yes | nc103 | 1.702609 | 191 | <400 |
| dmsite47 | 62919 | 63114 | P | TATATATATAA | 1.59 | 0.003 | yes | nc69 | 3.854612 | 195 | <400 |
| dmsite41 | 56525 | 56306 | N | TATAAATATGA | 1.11 | 0.273 | no | nc63 | 1.702609 | 219 | <400 |
| dmsite24 | 36165 | 36400 | P | TATAGAAATGA | 1.11 | 0.265 | no | nc30 | 3.439575 | 235 | <400 |
| dmsite40 | 56269 | 56018 | N | TATATAAATAA | 1.90 | 2.49e-05 | yes | nc61 | 4.17654 | 251 | <400 |
| dmsite54 | 72161 | 71905 | N | TATAATCATAA | 1.65 | 0.001 | yes | nc78 | 5.024537 | 256 | <400 |
| dmsite46 | 62818 | 63114 | P | TATAGGAATAA | 1.17 | 0.155 | no | nc69 | 3.854612 | 296 | <400 |
| dmsite48 | 62926 | 62606 | N | TATATATATAA | 1.57 | 0.005 | yes | nc68 | 3.439575 | 320 | <400 |
| dmsite92 | 146689 | 147068 | P | TATATGCATCA | 1.19 | 0.098 | no | nc136 | 1.702609 | 379 | <400 |
| dmsite59 | 75801 | 75406 | N | TATATGAATGA | 1.42 | 0.028 | no | nc83 | 1.702609 | 395 | <400 |
| dmsite78 | 116400 | 116816 | P | TATACATATTA | 1.56 | 0.010 | yes | nc104 | 1.854612 | 416 | <500 |
| dmsite4 | 6196 | 6617 | P | TATATCTATAA | 1.69 | 0.036 | no | nc5 | 7.635972 | 421 | <500 |
| dmsite2 | 2786 | 3257 | P | TATAGAAATAA | 1.47 | 0.019 | no | nc3 | 3.854612 | 471 | <500 |
| dmsite55 | 73231 | 72749 | N | TATAATCATAA | 1.12 | 0.255 | no | nc80 | 4.439575 | 482 | <500 |
| dmsite31 | 44805 | 44309 | N | TATATCAATGA | 1.09 | 0.292 | no | nc38 | 5.024537 | 496 | <500 |
| dmsite38 | 53665 | 54170 | P | TATAAATATTA | 0.87 | 0.794 | no | nc59 | 1.854612 | 505 | 500<1000 |
| dmsite14 | 20960 | 21473 | P | TATATTTATTA | 1.26 | 0.067 | no | nc18 | 1.854612 | 513 | 500<1000 |
| dmsite8 | 8405 | 7869 | N | TATAGAAATAA | 1.22 | 0.103 | no | nc6 | 5.024537 | 536 | 500<1000 |
| dmsite65 | 83344 | 83907 | P | TATATATATGA | 1.05 | 0.362 | no | nc90 | 5.024537 | 563 | 500<1000 |
| dmsite91 | 141427 | 142008 | P | TATATACATAA | 1.76 | 1.57e-06 | yes | nc126 | 1.702609 | 581 | 500<1000 |
| dmsite44 | 58592 | 57994 | N | TATACTAATTA | 1.18 | 0.189 | no | nc66 | 1.702609 | 598 | 500<1000 |
| dmsite11 | 12475 | 13082 | P | TATATCGATAA | 1.22 | 0.130 | no | nc10 | 1.854612 | 607 | 500<1000 |
| dmsite34 | 48510 | 49174 | P | TATACGTATAA | 1.46 | 0.011 | no | nc48 | 1.854612 | 664 | 500<1000 |
| dmsite35 | 51193 | 51910 | P | TATACTCATTA | 0.55 | 0.998 | no | nc52 | 3.854612 | 717 | 500<1000 |
| dmsite9 | 8643 | 7869 | N | TATAATAATAA | 1.54 | 0.005 | yes | nc6 | 5.024537 | 774 | 500<1000 |
| dmsite36 | 51482 | 50658 | N | TATATACATGA | 1.64 | 0.004 | yes | nc51 | 2.854612 | 824 | 500<1000 |
| dmsite23 | 35060 | 34229 | N | TATATGGATAA | 1.40 | 0.012 | no | nc28 | 3.439575 | 831 | 500<1000 |
| dmsite62 | 81954 | 82845 | P | TATATATATCA | 0.94 | 0.597 | no | nc89 | 4.661967 | 891 | 500<1000 |
| dmsite37 | 51587 | 50658 | N | TATAAATATCA | 1.84 | 0.001 | yes | nc51 | 2.854612 | 929 | 500<1000 |
| dmsite58 | 75028 | 73853 | N | TATATTTATAA | 1.52 | 0.009 | yes | nc82 | 5.314044 | 1175 | 1000-5000 |
| dmsite60 | 76735 | 75406 | N | TATATACATTA | 1.16 | 0.163 | no | nc83 | 1.702609 | 1329 | 1000-5000 |
| dmsite33 | 45816 | 44309 | N | TATATATATAA | 1.51 | 0.006 | yes | nc38 | 5.024537 | 1507 | 1000-5000 |
| dmsite16 | 28040 | 29689 | P | TATAGAGATTA | 1.07 | 0.324 | no | nc22 | 4.17654 | 1649 | 1000-5000 |
| dmsite18 | 30638 | 28948 | N | TATATATATAA | 1.56 | 0.007 | yes | nc21 | 2.854612 | 1690 | 1000-5000 |
| dmsite19 | 30647 | 28948 | N | TATATATATTA | 0.76 | 0.942 | no | nc21 | 2.854612 | 1699 | 1000-5000 |
| dmsite39 | 54369 | 56088 | P | TATATATATGA | 1.29 | 0.140 | no | nc62 | 3.854612 | 1719 | 1000-5000 |
| dmsite13 | 19705 | 21473 | P | TATATATATCA | 1.23 | 0.091 | no | nc18 | 1.854612 | 1768 | 1000-5000 |
| dmsite25 | 37705 | 35803 | N | TATACCCATCA | 1.36 | 0.023 | no | nc29 | 3.439575 | 1902 | 1000-5000 |
| dmsite57 | 75017 | 77012 | P | TATAAATATAA | 2.07 | 6.64e-05 | yes | nc84 | 1.854612 | 1995 | 1000-5000 |
| dmsite10 | 11085 | 13082 | P | TATAGGGATCA | 1.54 | 0.007 | yes | nc10 | 1.854612 | 1997 | 1000-5000 |
| dmsite21 | 30958 | 28948 | N | TATAAGCATGA | 0.90 | 0.777 | no | nc21 | 2.854612 | 2010 | 1000-5000 |
| dmsite32 | 45805 | 47866 | P | TATATATATAA | 1.67 | 0.004 | yes | nc46 | 1.854612 | 2061 | 1000-5000 |
| dmsite51 | 67554 | 70399 | P | TATATAAATCA | 1.22 | 0.165 | no | nc75 | 1.702609 | 2845 | 1000-5000 |
| dmsite75 | 110841 | 113867 | P | TATACGAATGA | 0.91 | 0.714 | no | nc103 | 1.702609 | 3026 | 1000-5000 |
| dmsite52 | 70455 | 67168 | N | TATATTTATGA | 1.66 | 0.001 | yes | nc74 | 2.854612 | 3287 | 1000-5000 |
| dmsite26 | 37943 | 41278 | P | TATACATATGA | 1.18 | 0.141 | no | nc34 | 1.702609 | 3335 | 1000-5000 |
| dmsite53 | 70543 | 67168 | N | TATATCAATTA | 0.96 | 0.604 | no | nc74 | 2.854612 | 3375 | 1000-5000 |
| dmsite56 | 73550 | 77012 | P | TATAAGAATAA | 1.03 | 0.419 | no | nc84 | 1.854612 | 3462 | 1000-5000 |
| dmsite83 | 119337 | 122874 | P | TATATCAATTA | 1.03 | 0.417 | no | nc109 | 1.854612 | 3537 | 1000-5000 |
| dmsite61 | 79253 | 75406 | N | TATATTCATAA | 2.07 | 5.44e-05 | yes | nc83 | 1.702609 | 3847 | 1000-5000 |
| dmsite27 | 39740 | 35803 | N | TATATTCATAA | 1.50 | 0.008 | yes | nc29 | 3.439575 | 3937 | 1000-5000 |
| dmsite70 | 106078 | 110141 | P | TATATAGATAA | 1.10 | 0.267 | no | nc99 | 2.854612 | 4063 | 1000-5000 |
| dmsite43 | 58587 | 63114 | P | TATAAATATAA | 1.59 | 0.016 | no | nc69 | 3.854612 | 4527 | 1000-5000 |
| dmsite50 | 65784 | 70399 | P | TATAATAATAA | 1.59 | 0.003 | yes | nc75 | 1.702609 | 4615 | 1000-5000 |
| dmsite15 | 24920 | 29689 | P | TATATAAATGA | 1.54 | 0.014 | no | nc22 | 4.17654 | 4769 | 1000-5000 |
| dmsite22 | 31367 | 36400 | P | TATAGCTATCA | 1.41 | 0.033 | no | nc30 | 3.439575 | 5033 | >5000 |
| dmsite49 | 65056 | 70399 | P | TATAGATATTA | 0.95 | 0.634 | no | nc75 | 1.702609 | 5343 | >5000 |
| dmsite20 | 30953 | 36400 | P | TATATGAATAA | 0.92 | 0.688 | no | nc30 | 3.439575 | 5447 | >5000 |
| dmsite28 | 41487 | 35803 | N | TATATCAATAA | 1.24 | 0.096 | no | nc29 | 3.439575 | 5684 | >5000 |
| dmsite17 | 30627 | 36400 | P | TATATATATAA | 1.55 | 0.005 | yes | nc30 | 3.439575 | 5773 | >5000 |
| dmsite42 | 57082 | 63114 | P | TATATGAATAA | 0.90 | 0.753 | no | nc69 | 3.854612 | 6032 | >5000 |
| dmsite67 | 85573 | 92095 | P | TATATGGATGA | 1.45 | 0.002 | yes | nc92 | 6.17654 | 6522 | >5000 |
| dmsite29 | 42654 | 35803 | N | TATATTCATAA | 1.40 | 0.025 | no | nc29 | 3.439575 | 6851 | >5000 |
| dmsite66 | 85239 | 92095 | P | TATACCTATGA | 1.32 | 0.019 | no | nc92 | 6.17654 | 6856 | >5000 |
| dmsite63 | 82840 | 75406 | N | TATAGTGATAA | 1.04 | 0.393 | no | nc83 | 1.702609 | 7434 | >5000 |
| dmsite64 | 83334 | 75406 | N | TATAGATATCA | 1.24 | 0.221 | no | nc83 | 1.702609 | 7928 | >5000 |
| dmsite12 | 16807 | 7869 | N | TATAGAAATTA | 1.53 | 0.004 | yes | nc6 | 5.024537 | 8938 | >5000 |
| dmsite68 | 87342 | 75406 | N | TATATGCATCA | 1.85 | 0.081 | no | nc83 | 1.702609 | 11936 | >5000 |
| dmsite69 | 92604 | 75406 | N | TATATACATAA | 1.82 | 0.118 | no | nc83 | 1.702609 | 17198 | >5000 |
| dmsite71 | 109775 | 75406 | N | TATAATAATGA | 1.30 | 0.060 | no | nc83 | 1.702609 | 34369 | >5000 |
| dmsite73 | 110096 | 75406 | N | TATAGTGATAA | 1.68 | 0.003 | yes | nc83 | 1.702609 | 34690 | >5000 |
| dmsite74 | 110277 | 75406 | N | TATAGTCATGA | 1.92 | 0.001 | yes | nc83 | 1.702609 | 34871 | >5000 |
| dmsite6 | 7150 | 112844 | N | TATATGTATCA | 1.75 | 0.005 | yes | nc102 | 1.854612 | 105694 | >5000 |
| dmsite5 | 6470 | 112844 | N | TATAAAGATCA | 1.75 | 0.040 | no | nc102 | 1.854612 | 106374 | >5000 |
| dmsite3 | 3803 | 112844 | N | TATATTGATAA | 1.61 | 0.003 | yes | nc102 | 1.854612 | 109041 | >5000 |
| dmsite1 | 117 | 112844 | N | TATAAATATCA | 1.61 | 0.007 | yes | nc102 | 1.854612 | 112727 | >5000 |

**Table S7 Analysis of the effect of DNA modification at the CPGDMM1 motif on the abundance of ncRNA using ANOVA.**

| Source | DF | Sum of Squares | Mean square | F Ratio | Prob > F |
| --- | --- | --- | --- | --- | --- |
| Modified | 1 | 11.23 | 11.23 | 23.13 | 0.0002 |
| Error | 15 | 7.28 | 0.48 | . |  |
| C.Toal | 16 | 18.52 |  |  |  |

**Supporting Figures**

Figure S1. Characteristics of SMRT sequencing results: (A) length distribution of sequence reads; (B) depth of coverage across the assembled chloroplast genome; and (C) abundance of various lengths of reads mapped to the chloroplast genome.


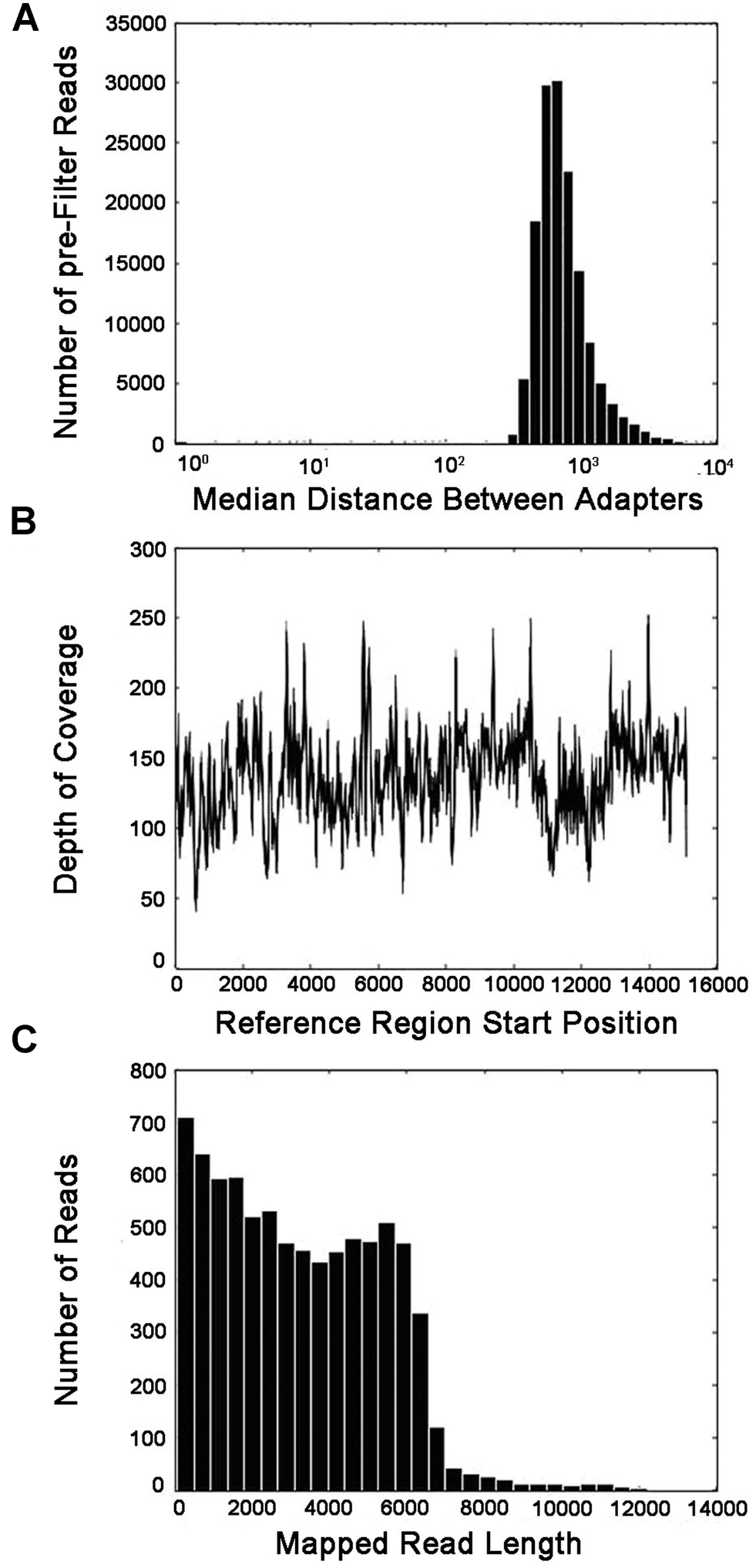

Supplement: File S1 — Contains the files: Figure S1. Characteristics of SMRT sequencing results: (A) length distribution of sequence reads; (B) depth of coverage across the assembled chloroplast genome; and (C) abundance of various lengths of reads mapped to the chloroplast genome. Table S1. Primers used for sequence assembly validation. Table S2. Primer sets used for strand-specific real-time qPCR. Table S3. Statistical testing of RNA-Seq and ss-qPCR results using one-sample t test. Table S4. Detailed information on motif CPGDMM1. Table S5. Detailed information on motif CPGDMM2. Table S6. Associations between DNA modification site (DMS) and downstream ncRNA expression. Table S7. Analysis of the effect of DNA modification at the CPGDMM1 motif on the abundance of ncRNA using ANOVA. (DOC) [file pone.0099314.s001.doc]
